# Mismatch repair-proficient tumor footprints in the sands of immune desert: mechanistic constraints and precision platforms

**DOI:** 10.3389/fimmu.2024.1414376

**Published:** 2024-07-19

**Authors:** Biswanath Majumder, Nishanth Belugali Nataraj, Leela Maitreyi, Santanu Datta

**Affiliations:** Bugworks Research India Pvt. Ltd., CCAMP, NCBS, Bangalore, India

**Keywords:** colorectal cancer, MMRp, tumor immune microenvironment, gut microbiota, therapeutic vulnerability, predictive biomarkers, precision medicine, functional platforms

## Abstract

Mismatch repair proficient (MMRp) tumors of colorectal origin are one of the prevalent yet unpredictable clinical challenges. Despite earnest efforts, optimal treatment modalities have yet to emerge for this class. The poor prognosis and limited actionability of MMRp are ascribed to a low neoantigen burden and a desert-like microenvironment. This review focuses on the critical roadblocks orchestrated by an immune evasive mechanistic milieu in the context of MMRp. The low density of effector immune cells, their weak spatiotemporal underpinnings, and the high-handedness of the IL-17-TGF-β signaling are intertwined and present formidable challenges for the existing therapies. Microbiome niche decorated by *Fusobacterium nucleatum* alters the metabolic program to maintain an immunosuppressive state. We also highlight the evolving strategies to repolarize and reinvigorate this microenvironment. Reconstruction of anti-tumor chemokine signaling, rational drug combinations eliciting T cell activation, and reprograming the maladapted microbiome are exciting developments in this direction. Alternative vulnerability of other DNA damage repair pathways is gaining momentum. Integration of liquid biopsy and ex vivo functional platforms provide precision oncology insights. We illustrated the perspectives and changing landscape of MMRp-CRC. The emerging opportunities discussed in this review can turn the tide in favor of fighting the treatment dilemma for this elusive cancer.

## 1 Introduction

One and half decades after the ‘immuno-oncology tsunami’ that hit the clinical development landscape and shifted the momentum of treatment modalities, multiple new immunotherapy agents are now on the horizon. This progress raised optimism for many late-stage cancers for which treatment options are heavily exhausted. However, a second breakthrough remains elusive. The overall response rate (ORR) also remains static (20–40%) for all cancers. A rapid surge of therapeutic targets for developing new checkpoint blockades and other immune-agonist classes is on the ground (1, 2). There is also a new wave of developing RNA-based cancer vaccines to provide deep, durable memory responses (3). One vaccine candidate in recent time showed encouraging outcomes in lethal pancreatic adenocarcinoma (4). Aligned with this momentum, we witness an explosion of combination trials that aim to enable better survival outcomes (5, 6).

Colorectal cancer (CRC) is considered a global epidemic; over the decades, it has emerged as a pivotal cancer type affecting the younger population and is associated with late-stage detection and poor overall survival (OS) (7). CRC individuals with DNA mismatch repair deficiency (MMRd) positively respond to immune checkpoint blockade (ICB). A striking response failure in mismatch repair-proficient (MMRp) patients shows the diminishing return of the same therapy. In CRC, most of the success stories for ICB, thus far, have been limited only to tumors that manifest MMRd. However, this subtype is represented by only 15% of all CRCs and is far behind endometrial cancers (30%) and gastric cancers (20%) (8). For MMRp, ORR is not very different (10–15%) between TMB high and low CRC (9, 10). This realization prompted looking for effective treatment options for the MMRp subtype. The trend implies the necessity of addressing unmet needs both at personalized and population levels. At the heart of this challenge is the underlying complexity of the tumor microenvironment and its unpredictable dynamic immune milieu that form a barrier to effective therapy (11).

In this review, we discuss the changing clinical landscape of MMRp-dependent cancer indications (mainly CRC) and their uniquely hostile tumor microenvironment that hinders the success of current immune-based interventions. Both conceptual progress and clinical translation are illustrated in the light of rapidly evolving spatial biology contexts like tertiary lymphoid structures and gut microbiomes. We discuss the perspectives and challenges of biomarker-guided treatment selections for MMRp agonist cancers. We also highlight the alternatively actionable DNA repair pathways as emerging vulnerabilities to combat the treatment dilemma. Finally, we presented significant progress on the horizon of patient-derived functional ex vivo platforms that raised the hope of bridging the critical mechanistic gaps between drug pipelines and informed clinical decisions.

## 2 Molecular alterations defining MMRp evolutionary trajectory

A pan-genomic analysis from the 100000 Genome Cancer Program integrating genomic and clinical data revealed the highest enrichment of specific DNA. It deciphered MMR signatures in MSI-high (i.e. MMRd) colon adenocarcinoma and uterine corpus endometrial carcinoma. MMRp in this spectrum showed a negative association with survival compared to MMRd patients. Germline variants of MMR in this study found their link with the onset of colon adenocarcinoma at an early age (12).

### 2.1 Facets of intratumor heterogeneity

The intertwining intratumor heterogeneity (ITH) with TMB and TILs contextually represents a complex biology. ITH, either primary, adaptive or acquired during treatments, is considered a spatiotemporal bottleneck for a high response rate and duration of response. ITH encompasses genetic, phenotypic and dynamic tumor microenvironmental milieu and orchestrates therapy resistance. It also leads to the evolution of new resistant clones or the expansion of drug-tolerant persisters (13). Deciphering this ITH through the lens of MMRp and developing strategies to combat ITH mutations in tolerant cells are vital for adopting rational intervention. The study in the autochthonous mice model of lung and colon cancers highlighted that high TMB and MMRd do not guarantee immunogenic tumor infiltrating lymphocytes (TILs) and a positive response to checkpoint blockade. The subclonal escape of T cell response in these tumors was orchestrated by an immune-mediated increase in clonal diversity (14).

Instead of relying explicitly on genomics, the gradient of TME modulators like chemokines, neoangiogenesis, blood vessels, nutrients, and oxygen, along with ECM stiffness in time and space, play pivotal roles (15–17). Echoing this realization, the immune milieu is thought to collectively provide an actionable dynamic niche that interacts with the drugs and make the tumors reactive to ICB (18, 19). CRC show properties of reversible (mutation-independent) drug tolerance where recurrence is imminent after tumor cells are relieved from therapy pressure. Interestingly, barcoding and mathematical modeling suggested that equipotent clonal complexity is maintained for all cells throughout this process without any temporospatial loss. Under such conditions, tumors mimic a developmentally programmed diapause state at transcriptomic and signaling levels to overcome environmental turbulence (20). Indeed, drug-tolerant and disseminating tumor cells are, in general, notorious immune evaders that take advantage of being unnoticed by the immune radar to escape the primary sites and survive as silent perpetrators (21, 22).

### 2.2 MMRp clonal heterogeneity: more than a binary class

Although MMRp and MMRd are binary molecular classes, recent profiling identified an intermediate category, i.e. heterogenous MMR or MMRh. The clonal overlap of MMRp and MMRd distinguishes it from the two classical subtypes. Gene expression analysis of CRC identified 14.5% of MMRd and 4.5% of MMRp cases as shared with this MMRh. The MMRh subclass allegedly evolves from double MMR gene loss. It is mechanistically linked to high TMB, TILs, and CD8 exhaustion phenotypes. High TMB (70 mut/mb) is attributable to higher subclonal variants. Genes associated with the MAPK pathway, antigen presentation and IFN-γ signaling pathway were significantly upregulated in MMRh class compared to MMRp (23). Moreover, 6-thioguanine and TMZ-induced enrichment of MMRd clones in MMRp tumors yielded encouraging outcomes. In two isogenic mice CT26 cell lines of MMRp (Mlh1+/+) and MMRd (Mlh1-/-) backgrounds, cross-complementing MMRp tumors selectively with MMRd clones rescued the immune surveillance program. MMRp clones, challenged with at least 50% MMRd cells, elicited tumor rejection. Both chemical induction and clonal competition strategies were able to underpin a heterogeneous MMR context of improved anti-tumor immune reactivity (24). This study in mice cell lines of CT-26 with MMRp backbone affirmed that reconstitution of MMRp clones with MMRd powered them to eliminate the MMRp fraction. The clonal and sub-clonal contexts of these two studies highlighted the differences in experimental approaches and interpretations of results. Specific TMB/neoantigens low subclones of mice tumors can evade an immune attack due to defects in cross-priming or active interference by dysfunctional T cells or immune ignorance. Moreover, these tumors are thought to acquire immunogenicity during *in vivo* repropagation at the clonal level but not at the sub-clonal level. As a result, the rapid contraction of MMRp clones was attainable (25, 26). This divergent clonal journey revealed the dynamicity of the ecological and evolutionary landscape of MMRp (27).

### 2.3 Altered gene regulation and mutations in MMRp

Further dissection of MMR status at the molecular level sheds light on the key regulatory elements driving transcriptomic machinery. Mutations (indels) in diverse CRC samples revealed that MSI-high CRC largely harbor gained enhancers that selectively offer the privilege of recurrent growth of these tumors through increased affinity for putative transcription factor, e.g. Forkhead Box D4 (FOXD4) and target gene overexpression that is regulated by these enhancers (28). Some of these genes have been implicated in chemoresistance, unrestricted oncogenic EGFR signaling, regulation of proliferation and apoptosis in primary CRC tumors and in established MSI cell lines. In the MSS cohort, the occurrence of enhancer indels was found to be at a much lower rate. Compared to MSS, MSI-high CRC has shown 50% more gained enhancers at TGTTT(Tn). It was linked to H3K27ac enrichment. A panel of 10 different FOX- transcription factors (FOX-TFs), encompassing FOXP2, FOXC1, FOXD3, FOXM1, FOXJ3, FOXA1, FOXO1, FOXO3, FOXG1 and FOXA2, presented the consensus sequence, a signature motif at indel alleles, and confirmed the binding affinity of FOXs. However, due to the degenerative nature of this consensus motif, findings did not specify the dominance of any single FOX member from the family in the enhancer activation. Instead, it proposed additional studies to fill this gap, elucidate cooperative interaction with other factors and inter-tumor heterogeneity, if any (29). This also implies the need for further delineation of other enhancers and super-enhancers to gauge their differential impact on the oncogenic driver alterations, making MMRd and MMRp tumors more vulnerable to therapies (30).

About 30% CRC are hereditary and germline predisposition affects CRC susceptibility. About 5%–7% of CRC cases are caused by germline mutations. Classic hereditary CRC syndromes are mainly due to germline mutations in APC, MUTYH, and mutations in genes encoding four mismatch repair enzymes, namely MSH2, MSH6, PMS2 and MLH1 (31, 32). Pathogenic somatic mutations (predominantly biallelic) in coding regions of one of these four mismatch repair enzymes lead to the development of MMR deficiency of CRC and eventually give rise to an MSI phenotype. Interestingly, Lynch syndrome (LS) or hereditary nonpolyposis, CRC, represents one-third of these MMRd, is an early onset CRC, therefore suggesting precedence of germline mutations (33). Similarly, tracking the germline defects in the MMRp genes provided significant screening opportunities for CRC of hereditary background. DNA mismatch repair protein O6-methylguanine DNA methyltransferase (MGMT) is frequently detected in CRC. Its epigenetic inactivation in somatic clusters is prevalent. However, elucidating whether the same defects are perpetuated in the germline background, particularly in MMRp, showed non-confirmatory results. No promoter methylation linked to constitutive MGMT inactivation was confirmed. Indeed, two rare heterozygous germline variants were detected in 4 families. Further segregation of these variants in neoplastic lesions in the affected family suggested that more data are needed to establish their link to MMRp in familial CRC (34). Although most CRCs by default are MSS, and MMRp represents low TMB, the study showed that 7.5% of colon and 9.5% of rectal cancers of this background also had high TMB (defined by >10 mutations per Mb). Interestingly, *KRAS* mutations and gene mutations involved in DNA damage repair (DDR) machinery and epigenetic modifiers were high in TMB-high MSS tumors. These findings suggest that molecular alterations are potential triggers of TMB in CRC (35). Like MMRh, these subsets open new opportunities for target dependency and vulnerabilities to gauge their promise in differentiated intervention.

## 3 Mechanistic constraints of MMRp tumors and strategies reversing them

**Figure 1:**
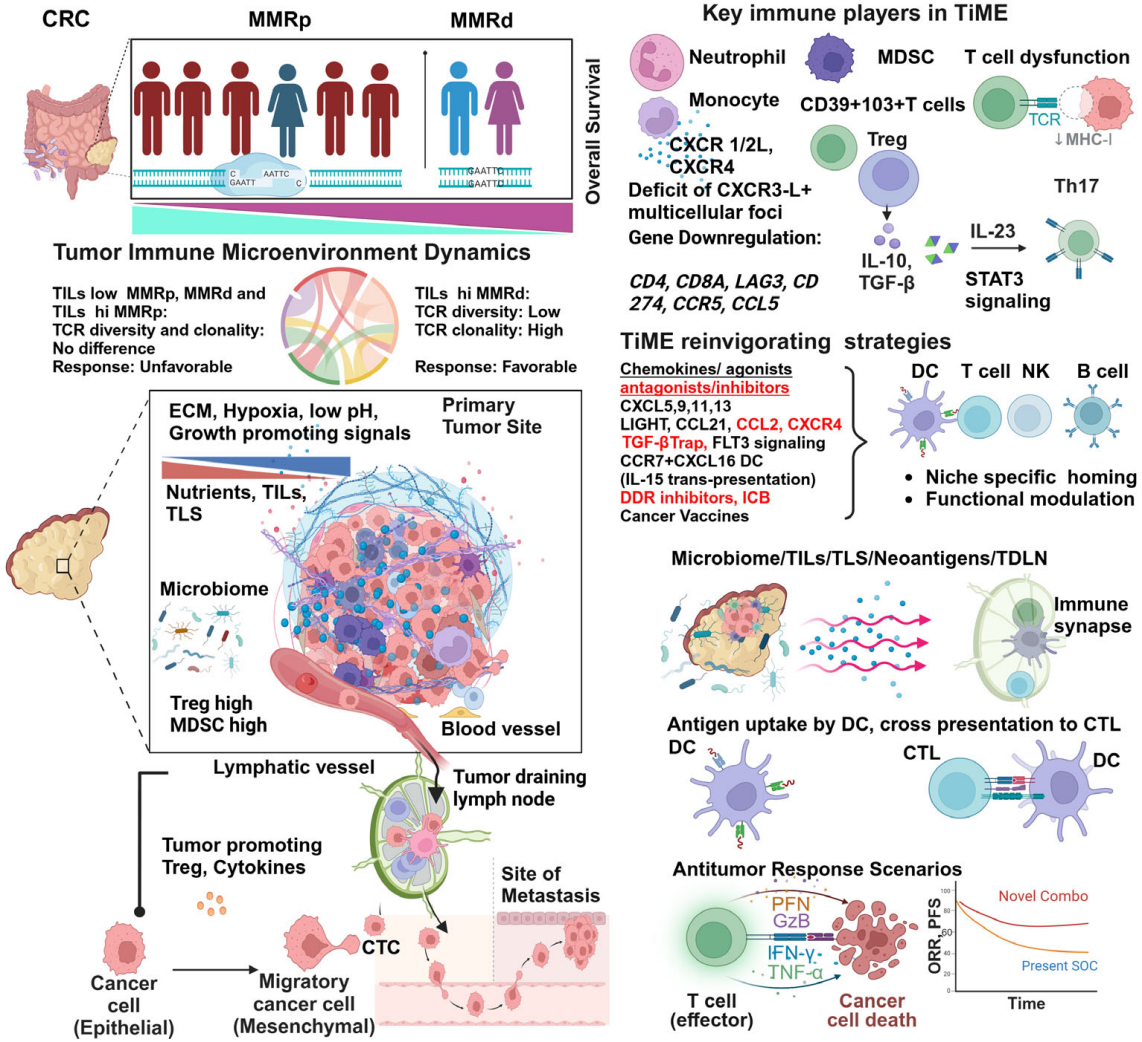
Tumor immune microenvironment of MMRp displays mechanistic constrains: potential strategies of reinvigorating. Despite representing more than three-fourths of the entire CRC population, the majority of MMRp CRC belongs to high-risk and poor prognoses. The TiME of CRC has multiple mechanistic barriers that hamper the therapy success. High TCR diversity and low TILs density in the margin and core at primary CRC sites are associated with desmoplastic stroma, growth-promoting oncogenic signaling, poor blood vessel density and patterns leading to oxygen and nutrients deprivation. Poor microbiome context and active involvement of Treg and MDSC in a traditionally low TMB milieu critically orchestrate immune evasion of disseminating tumor cells to distant sites through unguarded blood vessels and immunologically skewed TDLN’s surveillance. The phenotypic analysis of TILs confirms the presence of immune cell types of suppressive functions and corresponding cytokines and chemokine networks that protect the tumor from immune attacks. Several agonists and antagonists of chemokines, TGF-β-targeted therapy, and vaccines can act in concert with other strategies to reinvigorate and stabilize TILs and TLS via niche-specific recruitments of the anti-tumor immune army. Finally, augmenting neoantigen load and DC functionalities cross prime CD8+T cells. In totality, other immune and non-immune targets present in the TiME provide an opportunity to rationally target this challenging microenvironment in clinical settings and improve the response.

A hostile MMRp context of tumor-immune microenvironments (TiME) is a formidable therapeutic challenge in achieving clinical success. Low TMB and immunological ignorance are two critical hallmarks of the MMRp tumor ecosystem. Further complexity of a tangled network emanating from dysbiosis of microbiome and problematic metabolomics has the propensity to escalate the suppressiveness of the microenvironment to diverse therapy regimens. Understanding the dynamicity and dimensionality of this misdirected microenvironment and its immune restrictive milieu is pivotal to developing novel rationale interventions that can mount an all-out attack on these tumors (**Figure 1**).

### 3.1 TiME perspectives and TILs in MMRp

One critical differentiator in response to ICB between MMRd and MMRp is TILs density and its proximity (core and invasive margin) to target tumors. While a higher number of MMRp CRC patients are ascribed to be TILs deprived, i.e. they have lower TILs density, inter-tumor TILs heterogeneity (30–90%) in MMRd is not uncommon (**Figure 1**). This contexture includes a spatial heterogeneity of CD3+ and CD8+ TILs in the invasive margin and tumor core. Interestingly, a diminished TILs footprint in MMRd acts to underpin MMRp+ CRC response to ICB, a feature paralleling their functional TiME orientation (36).

#### 3.1.1 TCR diversity and clonal expansion

It is intriguing to note that not only the TILs density and distribution but TCR repertoire and clonal landscape in MMRp CRC are also different from its MMRd counterpart. While T cell clonality and the richness of TCR repertoire have similar imprints in MMRp with TILs low and TILs high context, a sharp contrast contradicts this signature in MMRd tumors. In MMRd, higher T cell clonality was observed to be matched with lower TCR richness in TILs high tumors while comparing them with TILs low tumors within the same MMR class (**Figure 1**). It is imperative to note that under high TMB, T cells are clonally expanded in MMRd with high TILs. The constraints of low TMB in MMRp made both TILs low and TILs high tumor uniform in their clonal expansion program, and they maintained similar TCR diversity (37). These findings show that T cell clonal dynamics in TMB with a low and high background may reciprocally impact immunosurveillance. Studies in other cancers claimed that global TMB alone is not a perfect proxy for the foreignness of antigens. An evolutionarily persistent TMB, due to single or multiple copy regions per cell, provides a better response opportunity by creating a bottleneck for tumors that is difficult for them to overcome (38).

#### 3.1.2 TILs numbers and spatial contexture

The organ specific immune atlas is emerging as an important platform to capture deeper phenotypic perspectives and insights. Single-cell RNA sequencing revealed the existence of a distinct immune hub in a spatially defined CRC cancer-immune network. A shared myeloid-rich inflammatory immune hub in tumors below the colonic lumen and CXCR3-ligand positive anti-tumor multicellular foci accompanied by activated T cells in MMRd tumors contextually distinguish them from MMRp (39). Like spatial TILs, quantitative analysis of global TILs in MMRp is a logical step in defining their prognostic impact. In high power field (HPF) quantification of TILs, based on a threshold set as >3 (high) vs <3 (low), five years of recurrence-free survival was observed to be higher in MMRp CRC with high TILs (94.6%) compared to their low TILs counterpart (77.9%). More importantly, in multivariate analysis using stages and TILs as key discriminators along with MMRp status, the higher stage with high TILs resulted in a similar relapse-free survival (RFS) to that of the lower stage alone without impacting OS (40).

### 3.2 TiME beyond TILs: APC defects, γδT and NK cells

Reactive TILs are not the only immune subset that encounters tumors. Multiple evasion points that perturb the TiME are contextually intertwined.

#### 3.2.1 PD1+γδT cells in B2M defective immune network

In the case of conventional antigen-presenting cells (APC), gene defects mainly govern β2 microglobulins (*B2M*) inactivation and HLA class 1 dysregulation in CRC. ICB under these circumstances increases γδT cell subsets with PD1, killer-cell immunoglobulin-like receptors (KIR) and cytotoxic activation markers in TME. These players, in concerts, determine the preferential retention of ICB responsiveness in cell lines of MMRd and MMRp backgrounds as well as patient-derived organoids that are defective of *B2M* gene and show concomitant loss of HLA class1 presentation machinery. HT-29 CRC cell lines of MMRp lineage retain B2M function compared to MMRd lines (HCT-15 and Lovo), where *B2M* gene defects (HLA -1 antigen presentation loss) in HT-15 cell lines instigate the most profound ICB response orchestrated mainly by PD1+ γδT cells in a coculture based drug reactivity assay (measuring Caspase3/7). The reintroduction of *B2M* genes in MMRd cell lines resulted in a loss of tumor killing by γδT cells in response to ICB under similar conditions. Further delineation of MMRd clinical CRC samples by multiplex spatial immune profiling suggested a remarkable increase in γδT cells in *B2M* defective cases. MMRd Patient-derived organoids with *B2M* loss elicited a better response by PD1+ restricted γδT cells (41).

#### 3.2.2 FLT3L signaling defects in DC functionality

Preclinical models of MMRp CRC (accounting for 95% of all mCRC) revealed that these tumors preferentially spread to the liver following orthotopic implantation but are restricted when heterotopically implanted in a subcutaneous site known for its context deficit and poor vascularization. This complementary liver metastasis model importantly recapitulated the paucity of CD8 and DC, consequently maintaining the non-responsiveness to immune checkpoint blockade (ICB). Combined treatment of Feline McDonough sarcoma (FMS)-like tyrosine kinase 3 ligand (Flt3L) plus ICB therapy improved survival by enhancing dendritic cell infiltration (42) (**Figure 1**). Indeed, whole-genome analyses of metastatic colorectal cancers from a pan-cancer Hartwig database of 2256 MMRp samples confirmed that only 1.6% of these samples clonally showed *B2M* and concurrent loss of heterogenicity (LOH), limiting their prognostication impact (43). In another study, pexidartinib, a CSF-1R–directed tyrosine kinase inhibitor (TKI), in combination with durvalumab (anti–PDL-1) in CRC and other cancers, resulted in limited efficacy. Pexidartinib impaired the development and functionalities of DCs due to the inhibition of FLT3 signaling ex vivo and *in vivo* (44). These findings illustrate the importance of maintaining active FLT3 signaling to achieve reasonable response (**Figure 1**).

#### 3.2.3 NK cells rescue B2M-driven immune dysfunction

Interestingly, in MMRd CRC, *B2M* mutations that canonically disrupt antigen presentation machinery showed paradoxical outcomes. It prevented disease recurrence, metastasis and helped manage prolonged survival. In this case, NK cell mediated inhibitory effects in the absence of HLA 1 defect prevented metastatic spread (45, 46). Unlike CRC, in endometrial cancer, defects in T cell activation signaling due to *JAK1* mutation turn the tumors immune inert (47). Phase I/II multicenter study of autologous DC with Avelumab in mCRC for pharmacodynamics (pD), safety and efficacy showed well-tolerated outcomes but a modest 6-month PFS (only for 11% of patients). Interestingly, the rewiring of lipid metabolism against glutamine and glucose utilization and the generating reactive oxygen species (ROS) in response to this combination contributed to longitudinal progression. There is an urgent call for tailoring novel therapies to target this dependency as a vulnerable checkpoint (48).

### 3.3 Tertiary lymphoid structure in MMRp is an elusive immune hub

In recent years, conceptual progress and clinical promises of tertiary lymphoid structures (TLS), a specialized tumor-immune microenvironmental niche, have attracted attention (49). They have a concerted influence on priming/amplification//licensing itineraries in TiME. The TLS army involves diverse lineage-specific subsets like plasma cells/B cells, different DCs like conventional DC (cDC), follicular DC (fDC) and other myeloid and lymphoid-derived cell types. In CRC, TIL, TLS and their abundance are mainly elucidated in MMRd (50). For MMRp, the ongoing efforts dissect niche-dependent immune evasion and design rationale therapies that can enrich the TLS footprint and redefine ICB response. In parallel, microbiome-immune crosstalk in eliciting anti-tumor response is appreciated in CRC and implicated the roles of TLS (51). From the qualitative and quantitative perspectives, the size, composition and spatiotemporal dynamics of TLS and non-TLS immune hubs like TILs and lymphonets promise new therapeutic modalities (52). A 56-marker multiplex IHC-driven cellular classifier (CODEX) at the invasive front identified CD4+PDL-1-positive cells in the granulocyte neighborhood as the only positive prognostic marker in high-risk advanced-stage CRC. In contrast, the lack of inter-compartment connectivity in TiME contributes to unfavorable outcomes (53). At preclinical levels, however, there are limitations to the potential human translation of TLS. One reason is that besides wide gaps in TME, mice tumors exhibit rapid and aggressive growth. This property inherently restricts the scope of mature TLS formation within a defined temporal neogenesis window. The cells that populate an immature or suppressive TLS, e.g., Breg, Treg and MDSC, can also perpetuate in the MMRp (54–56).

### 3.4 Chemokines and immunomodulators in homing and reinvigoration of MMRp

Chemokines, released by tumor cells and other cell types like stromal fibroblasts and endothelial cells, act as chemo-attractants. Through engaging cognate receptors, they recruit immune cells that are anti-tumor or immune suppressive (pro-tumor) in functions. Multiple interactive chemokine axes also influence therapy outcomes. These depend on the types of chemokine ligands, cognate receptors on the target cells and specific TME contexts (57, 58).

Mechanistically, the reconstituted chemokines milieu could be a critical orchestrator of reinvigorating the depressed TLS and TiME. Spatially delineated biomarkers or ‘biopatterns’ shed light on the niche-specific recruitment and interactions of immune cells in TME. A comprehensive knowledge of their contexts is important for microenvironment-guided therapy selection (58, 59). The MMRp tumor-immune niche is the home of several suppressive immune subsets. Monocytes, neutrophils, MDSC, Tregs and Th17 cells are lead players in this domain and are responsible for maintaining a tumor-friendly suppressive network (**Figure 1** and **Table 1**).

**Table 1:** Key Chemokines, their receptors, functions and combating strategies in MMRp CRC.

Chemokine Axis		Chemokines in MMRp CRC		Refs
Ligand	Receptor	Key findings of dysregulation	Combating strategies	
**CXCL 1,2,8**	**CXCR1/2**	1. Preferential recruitment of TAN, monocytes, Tregs, and IL-17. 2. Deprivation of CD8 and anti-tumor immune cells	1. Neoadjuvant intra-tumoral influenza vaccine: i) down regulates *CXCL1, CXCL2*, *CXCL8* and *CXCR2*, ii) downregulates genes related to neutrophils and its activation, iii) upregulates the transcription of *Th1*, *CD8*, *TIL*s, cytotoxic function, CD8+T cell infiltration, iv) increases CD8/neutrophil ratio, down regulates FOXP3 (spatial IHC). 2. Anti-IL-8, a monoclonal antibody HuMax-IL8 reduced serum IL8 and showed safety and stable disease in phase 1 trial.	(39, 60, 61)
CXCL5, CXCL6	CXCR 1, 2	Attraction of TAN and monocytes	1. Intra-tumoral influenza vaccine, i. decreases the expression of genes *CXCL5, CXCL6* (innate immune), ii. decreases *TLR1* and *TGF-β1*	(39, 60)
CXCL12	CXR4	1. In orthotopic CT26 and SL4, CXCR4 is expressed by MDSC like Ly6C-low (but not Ly6C-high) monocytes and Ly6G+neutrophils. 2. Anti-VEGFR2, upregulates CXCR4/CXCL12 and recruits these monocytes and neutrophils.	1. Blockade of CXCR4 by Plerixafor and selective Ly6C genetic depletion in monocytes rescues anti-VEGFR2 mediated delay in tumor growth.	(62)
CCL3, CCL4,**5**, CCL17	**CCR5**, CCR4 (for CCL17)	1. In CRCR, CCR5-CCL5 is associated with CMS 1 and CMS 4, high TMB, and high TILs, TIS, and low PDL-1 level. 2. High CCR5 and CCL5 transcripts maintain high ratio with M2 and Tregs. 3 In both MMRp and MMRd, *F. neuleatum* derived succinate reduces CCL5, CXCL10, IFN-β, desensitize CD8 to ICB 4. In CRC-L, MDSC via CXCL 9 and 10 triggers T cell movement in invasive margin, CCL5+CD4 and CD8 cells induces tumor-growth via macrophage MMP.	1. CCR5/CCL5 low group benefits from cetuximab and FOLFOX 2. GITR ligation by agonist on immune cells restores TILs, i) enhances CCL3, 4, CCL17, and CXCL9 levels in ex vivo TILs culture, ii) shows a trend to increase CCL3,4, CCL17, CXCL1, and CXCL5 levels in CRL-M, iii) induces TILs expansions, functionality and augments cytokines TNF-α and IFN-γ. 3. Disruption of microbiota-metabolite-immune- crosstalk re-sensitizes CD8 to ICB by i) FMT with low *F. nucletum* from responder, ii) lowering succinate by reducing Fn by metronidazole, iii) restoring cGAS-IFN-β dependent Th1 type CCL5 and CXCL10. 4. In rMMRp, CCR5 antagonist and maraviroc increased anti-tumoral chemokines during treatment 5. Maraviroc in CRC (liver met) ex vivo organotypic culture showed anti-tumor M1 polarization. 6. oHSV expressing a cetuximab-CCL5 fusion protein (OV-Cmab-CCL5) was able to trigger recruitment and activation of macrophages, CD8 and NK cells, shrink tumors and prolonged survival of mice. 7. In pretreated MMRp CRC, ADC vaccine with Avelumab, in long term survivor (n=1), showed >240-fold increase in CCL5 in serum.	(48, 63–66)
**CXCL9,10,**11, **CXCL13**	**CXCR3** **CXCR5** (for CXCL13)	1. MMRp differs from anti-tumor hub of MMRd characterized by CXCR3L+ multicellular foci, ISG+myeloid and malignant cells, activated T cells, CXCL13+T cells, IFN-γ+T cells, CXCL9–11, and upregulated CXCR3 in activated T cells and in DC). 2. MMRp-R type CRC at base line, CXCL13 high expression, PD1+CD8+ IFN-γ+TILs with TH17 low are responsive to ICB and resemble MMRd TILs	1. VSV-OV expressing CXCL-9 increases local CXCL9 level and chemokine gradient in culture but failed to attract ATC)	(15, 39)
CCL19, 21	**CCR7**	1. In MMRp-R, PD1+ TILs exhibit an exhausted/effector memory gene expression, lack IL-2, IL-15 and TNF-α, lower gene expression ofCD28, CCR7, IL-7R and CD62L PD1neg CD8 cells.		(67)

CRCR, CRC right sided; oHSV, Oncolytic herpes simplex virus type 1; CRCL, CRC Liver Met; TIS, T-cell inflammed score; ADC, autologous DC; ADT, Adoptive T cell Transfer; ISG, Interferon-Stimulated Genes; GITR, Glucocorticoid-Induced TNFR-Relate; VSV-OV, Vesicular stomatitis virus-Oncolytic virus.

#### 3.4.1 CXCR1/2, TGF-β signaling and Th17

Preferential recruitments of tumor-associated neutrophils (TAN), monocytes, other myeloid- derived suppressor cells (MDSCs), Tregs, and Th17 functionally cooperate through chemokines like CXCR1/2 and CXCR4-CXCL12. In this network, IL-10, TGF-β, IL-10, IL-23, and STAT3 signaling worsen the prognosis (58, 67–70). Their interplay hampers the prospects of therapies. For example, anti-VEGFR treatment in MMRp mice orthotopic model triggers a positive feedback loop that further upregulates CXCR4-CCL12 and recruit monocytes and neutrophils in the TME (62).

#### 3.4.2 Critical regulation dynamics of CCR5-CCL5 axis

In a hyperpolarized TME, CCR5 and CCL5 in MMRp are tightly associated with the right-side colon, poor prognosis-related consensus molecular subtypes 1 and 4 (CMS 1 and CMS 4), high TMB, and high TILs. Different myeloid and lymphoid subsets like M1, M2 macrophages, B cells, CD4, CD8, T regs and NK cells, in coordination with PDL-1, CTLA-4 and PARP, orchestrate a depressed immune network. It is evident that CCR5/CCL5 low group benefits from targeted therapy of cetuximab and FOLFOX (71). Paradoxically, past research showed a poor prognostic link to CCR5. The study showed a marginal improvement in specific combinations. However, due to the unavailability of data from retrospective analysis, confounding effects cannot be ruled out (71). Collectively, these modalities can guide the homing of immune cells in MMRp tumors known for traditionally lacking reactive TLS footprints. As illustrated in **Figure 1** and **Table 1**, TiME of MMRp selective tumors has deficits in TILs and TLS. It shows a distinct bias for Treg and MDSC (mainly monocytes and TAN). Moreover, the immunosuppressive cytokines that MDSCs augment, facilitate epithelial-mesenchymal transition (EMT), increase the propensity for distant metastasis of disseminated tumor cells and confer failure to therapies (67, 72, 73). In the invasive margin of CRC liver metastasis (CRC-L), CCL5+CD4 and CD8 cells, recruited by myeloid-derived CXCL9 and CXCL10, attract macrophages promoting invasion and tumor growth by MMP (65). However, the same CCL5 in T cells is impaired under succinate high microenvironment created by *F. nucleatum* and mediate ICB nonresponse (66). These findings suggest the contrasting roles of CCL5-CCR5 axis in different TME contexts.

#### 3.4.3 Chemokine agonists and antagonists in reshaping MMRp TiME

A number of therapeutic options are emerging on this horizon to fill the vacuum. The key modulators are chemokine agonists and antagonists that reciprocally orchestrate niche-specific recruitment and modulation dynamics. Functionally active chemokine axes, their dysregulation in MMRp and mechanistic interventions are illustrated in **Table 1**. A rational approach in MMRp can unlock the potential of reinvigorating its TiME. Retaining or reconstructing niche specific chemokine networks led by A) CXCR3 and its ligands CXCL9, 10, 11, CXCR5 and its ligand CXCL13, B) CXCR2 and its ligand CXCL5,6, C) CCR4 and its ligand CCL17, and D: CCR5 and its ligands CCL5, and additionally CCR7 hold promise in this space (reviewed in 57,58). Chemokines like CCL1, CCL2, CCL8, CCL12, and their receptors (e.g. CXCR1/2, CXCR4) reciprocally facilitate the homing of myeloid suppressors (monocytes, TAN, MDSC) and Treg. The drugs or antagonists targeting their actions can rejuvenate the immune reactive interface (58, 65, 73).

Neoadjuvant intra-tumoral influenza vaccine in MMRp CRC showed downregulation of pro-tumor chemokine genes, TGF-β genes. It concomitantly upregulated genes involved in Th1, CD8, increased TILs and cytotoxic function. The same vaccine decreased the Treg transcription factor FOXP3 at the protein level (60). Autologous dendritic cell (ADC) vaccine with Avelumab showed a decline in serum CCL2 level in pretreated MMRp CRC and a 240-fold increase of serum CCL5 in a long-term survivor (48). Inhibition of CXCR4 by Plerixafor or selective Ly6C targeted genetic ablation in monocytes rescues mice from anti-VEGFR2 induced tumor progression (62). CCR5 antagonist maraviroc has been tested in preclinical ex vivo tumor culture. In an independent clinical study, it demonstrated anti-tumor macrophage repolarization and anti-tumor chemokine augmentation, respectively (65, 74). CXCR3/5 ligands selectively recruit cells like DC, CD8 and T helper 1 as part of a niche-specific homing program (39, 58, 75). Therapies rely on agonists that drive the targeted enrichment of chemokines like CXCL9, 10, 11 and 13 and facilitate CD8 recruitment. For example, Glucocorticoid-Induced TNFR-Related (GITR) ligation by its agonist enhanced CCL3, CCL4, CCL17, and CXCL9 levels in CRC derived TILs in ex vivo culture. It induced TILs expansions, functionality and augmented proinflammatory cytokine (e.g. TNF-α, IFN-γ) (64). Gut microbiota adds a layer of criticality to this interface. Disruption of microbiota-metabolite-immune- crosstalk with low *F. nucleatum* (Fn) from responder or reducing *F. nucleatum* by metronidazole diminished the local succinic acid in TME and re-sensitized CD8 to ICB. This intervention also restored cGAS-IFN-β dependent CCL5 and CXCL10 following their decrease by high succinate (66). Anti-IL-8 antibody reduced serum IL-8 in phase 1 trial (61). Oncolytic virus expressing CXCL-9 restored local chemokine gradient but failed to recruit adoptive T cells (ATC) in culture (15).

Chemokines function as important modulators of TLS in both MMRd, MMRp scenarios (76, 77). Chemokines like LIGHT, LTa, CCL21, and APC activating agonists for TLR4 and CD40 are critical druggable targets to boost TLS (78). CCR7+ CXCL16+DC mediated trans-presentation of IL-15 to effector-like CTLs in perivascular niches orchestrate their survival and expansion. This survival and proliferation signaling loop averts an irreversible terminal differentiation of CTLs into the hypofunctional or tolerant state and maximizes the quality of response (79), (**Figure 1** and **Table 1**).

IL-15 trans-presentation, TGF-β-Trap with anti-EGFR, DDR inhibitors and cancer vaccines are also under active development to overcome the outstanding challenges (48, 60, 79–81). While mechanistically compatible TILs in such scenarios may provide a milieu for immune-based interventions, other tumor intrinsic evasion strategies can still be a barrier that avert T cell-mediated attack of tumors (22). For example, perforins and granzymes are two critical polarized cytotoxic effector molecules released from activated NK and T cells. Perforins act as a port of entry for granzymes. However, tumor cells manipulate their inherent ESCRT-mediated membrane trafficking strategy to repair these pores and, therefore, block the entry of granzyme (82).

### 3.5 Lymph node niche and immune surveillance: TGF-β signaling intervention

At the systemic level, a compromised immune activation network signals a prospective disease that is often advanced beyond primary sites. A recent study also proposed that preserving the tumor-draining lymph nodes (TDLN) may benefit anti-tumor immune reactions. Based on CRC data, dissection of immune phenotypic profiling showed differentiated TILs and TCR repertoire dynamics in lymph nodes (LN). This profiling separated MMRd from MMRp. In general, lymph node lymphocytes (LNL) show an intermediate functional state when compared with peripheral blood (lowest) and intratumor TILs (richest in tumor-reactive TILs). Stage-dependent TIL analysis also showed higher TILs in early-stage MMRd compared to matched early-stage or late-stage MMRp. Cytotoxicity-related genes also maintain similar enrichment patterns in MSI-H/MMRd cohorts. In this continuum, shared TCRs analysis of TILs showed their lower percentage in the proximal LN (pLN) of MMRp compared to MMRd. These data show the potential benefit of avoiding excessive non-metastasis LN dissection in MMRd (83, 84). CRC from MMRp origin contains neoantigen reactive autologous TILs co-expressing CD39 + 103+ T cell subsets in the CMS4 (less immunogenic) context. These T cells are known for promoting a paracrine TGF-β signaling loop and have the worst prognosis. Further delineation of checkpoint status targeting TGF-β and its trap with PDL-1, in this context, expected to reinvigorate TIL effector functions (69, 85, 86). However, an anti-PD-L1:TGF-β trap fusion protein directly targeting MSS-positive metastatic CRC failed to control the recurrence of ctDNA and, instead, elevated the level of ctDNA (86). Other strategies of dual targeting TGF-β with EGFR (e.g. BCA 101) are under development. Its combination with ICB in preclinical *in vitro* coculture assay using PBMC in EGFR-insensitive human colon cancer cell line HCT-116 (MSI hi) showed synergy with a high TGF-β footprint. Immune-reconstituted human colon cancer HT-29 (MSS) in mice xenograft model mechanistically elicited a potent immune-mediated anti-tumor response upon BCA 101 exposure (80) and **Figure 1**. More studies intersecting the TGF-β crosstalk in suppressive MMRp are needed to boost the quality of responses. *KRAS* mutant CRC and similar cancers were portrayed as undruggable until recently. A lymph node-targeted KRAS mutant peptide vaccine with a CpG oligo adjuvant (Amph-CpG-7909) in the AMPIFY-201 trial tested this therapy on 5 CRC patients, all from MMRp background. In 84% of cases, it showed T cell response *ex vivo*. Of this response, 54% involved both CD4 and CD8-specific T cells. In 84% of cases, there was a decline in biomarker (ct-DNA level) from the baseline, and in 24% (3 Ca-Pancreas and 3 CRC) cases, total clearance of biomarkers was achieved (87) and **Figure 1**. One outstanding question is what should be an ideal therapy plan for CRC patients with preexisting autoimmune conditions. IL-17-IL-23 axis is a clinical target in multiple autoimmune disorders (88). Therefore, a rational combination of these agents in MMRp patients with existing and new I-O and non-IO agents deserves evaluation through proper trials.

## 4 Onco-microbiome and metabolome interface in MMRp tumors

Among different theories, a complex interplay of intrinsic and extrinsic factors and cellular plasticity governs the initiation, maintenance and progression of cancers (89). The cancer risk mapping in higher mammals identified new attributes independent of body size and age that were thought to accommodate more cancer-causing mutations or “bad luck” mutations, initially coined by Tomasetti and Vogelstein (90). These risks include diets and loss of the gut microbiome homeostasis (91). New insights highlighted the clouds of complex systemic landscape in the frontiers of cancer hallmark. Besides metabolic alteration, ageing and obesity, tissue macro and microenvironment, myeloid dysfunction and other physiological dysregulation, genetic and environmental factors, this conceptual progress reiterates microbiome as a lead dimension (92).

### 4.1 Tumor invading gut microbiota and its orientation in MMRp

In recent years, the role of the microbiome in redefining novel immune therapy has fascinated clinicians and researchers alike. Physiological decoding of the microbiome and its metabolite derivatives (e.g., amino acids and short-chain fatty acids) in MMRd and MMRp identified functionally distinct footprints. For example, enrichment of *Bacteroides fragilis* and sulfidogenic *Fusobacterium nucleatum (Fn)* were profound in MMRd. In contrast, *Bacteroides fragilis* was deprived in the MMRp tumor-microbiome interface. Both these species are linked to a maladapted metabolic landscape in the gut niche (93, 94). To further narrow down at the level of clades, a recent study identified that clade 2 of the Fn subspecies animalis (Fna) strain is predominant behind intra-tumoral loads following heavy colonization in the CRC niche (95)*. Fusobacterium nucleatum* gets the upper hand in an MMRp ecosystem. It confers resistance to chemotherapy, promotes Wnt signaling, binds to TIGIT through its Fap2 component, and activates inhibitory cytokine-producing Treg and M2 macrophages. TLR4-NFkB signaling under such conditions is impaired in chemo-resistant CRC due to the upregulation of autophagy and anti-apoptotic signals (96, 97). Among different metabolites, bacteria-derived inosine acts via A2A adenosine receptor (A2AR) in Th1 cells, facilitates T cell and DC crosstalk and increases the metabolic fitness of CD8+T cells to trigger tumor killing (98). It serves as an alternative carbon source where there is a restriction of glucose availability to CD8 (99). The chronic inflammation due to a low-fibre diet and altered bacterial interaction with archaea insults the gut ecosystem, shifting a balance to dysbiosis (100, 101). This property also orchestrated a metabolomics bias where MMRd tumor had more association with host protective amino acid biosynthesis (102).

### 4.2 Interplay of the microbiome and immune niche: TGF-β and Th17 paradigm

**Figure 2:**
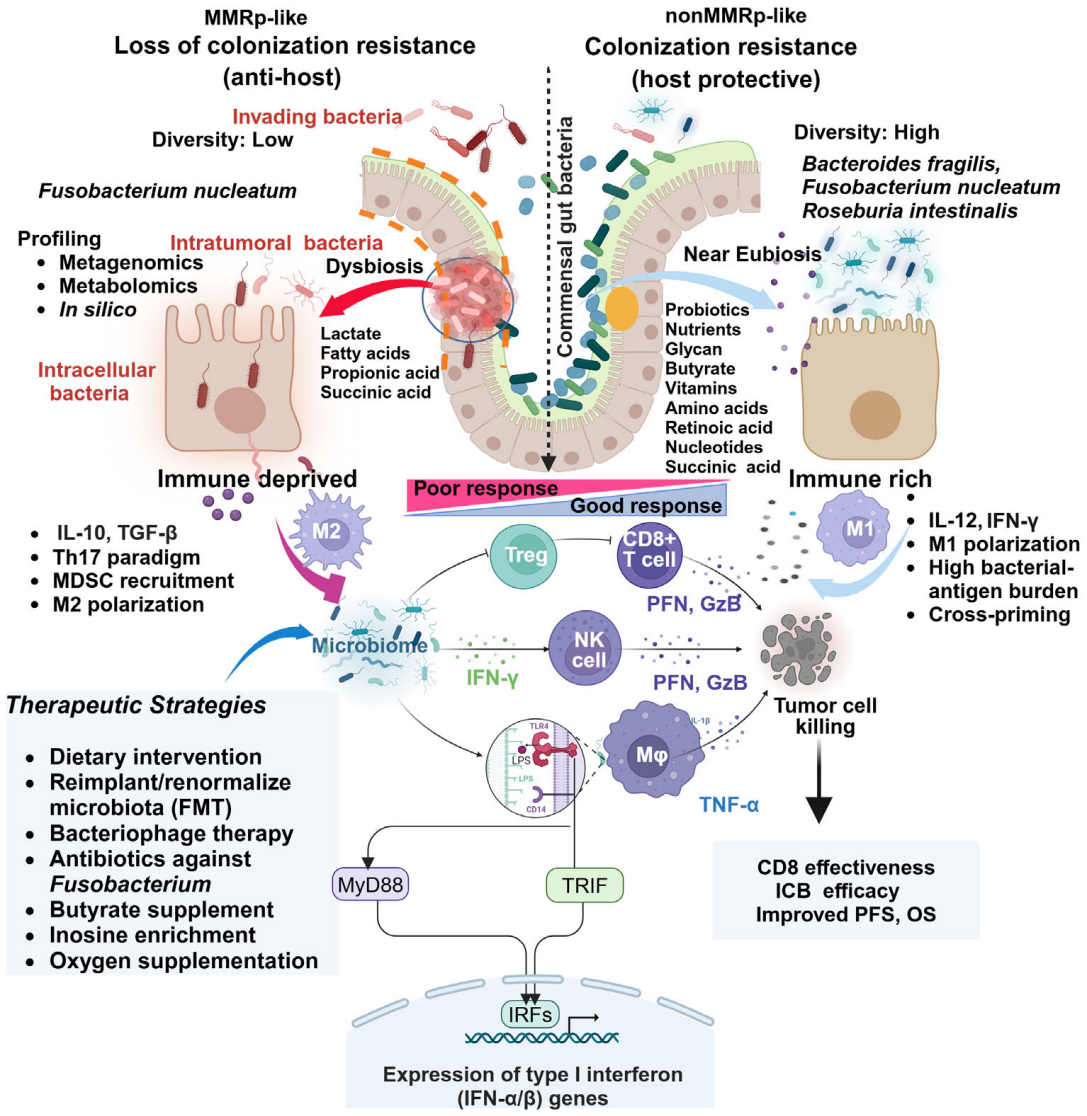
Comparative gut microbiome profiles in MMRp and non MMRp tumor hosts underline multiple contextual constraints and explain barriers to therapy success. The global loss of protective gut resident commensal bacteria makes the border porous for invading bacteria and supports their colonization. Different next-generation sequencing platforms and *in silico* analysis enable determining the high load of such bacteria and dissecting the loss of diversity in MMRp interface. This maladaptation promotes the metabolic bias in the microenvironment characterized by the overproduction of lactate, propionate, long-chain fatty acids and concomitant loss of glycans, short-chain fatty acids like butyrates, vitamins, amino acids, retinoic acids, and nucleic acids. In such conditions, intra-tumoral and intracellular bacteria facilitate the polarization of immune cells like MDSC, Treg and M2 macrophages, creating a suppressive paracrine cytokine loop. This polarization indicates a sharp contrast with MMRd, where a permissive metabolic footprint favors the preservation of cytokines like IL-12 and IFN-γ, bacterial antigen presentation by M1 macrophages. Under this condition, the interaction of bacterial LPS with TLR in macrophages triggers a signaling pathway via canonical myeloid differentiation primary response 88 (MyD88) and TIR domain-containing adaptor inducing interferon-β (TRIF) that engages IRFs and produces type 1 IFN. Fn-mediated altered cytokines and other anti-apoptotic mechanisms confer resistance by orchestrating an M1 toM2 paradigm shift. Macrophages (M1), CD8, and NK-mediated production of anti-tumor cytotoxic effectors like perforin (PFN) and granzyme-B (GzB) elicit tumor-killing effects. TGF-β, IL-10 and IL-17 impair immune-effector function in MMRp. Multiple strategies focusing on improving the hostile tumor-microbiome interface in MMRp can reverse the suppressive state. Adapted from “Keystone Gut Microbiota Species Provide Colonization Resistance to Invading Bacteria” by BioRender.com (2021). Retrieved from, https://app.biorender.com/biorender-templates.

Loss of resistance to the colonization of harmful invading bacteria, a new hub created by them in the vicinity of the tumor and inside tumor core including immune cells challenge the host protective anti-tumor immune function. Th17 cells help maintain homeostasis (eubiosis) in a normal gut. However, damage caused by bacterial invasion on gut epithelia triggers the loss of IL-17RA. The systemic spread of Th17 cells and B cells to distal organs facilitates tumor promotion via Dual oxidase 2 (DUOX2). This study showed compartmentalized and context-dependent roles of IL-17 signaling (103). Specific cellular contexts of the IL-1 receptor (IL-1R) also determine the impact of microbial induced IL1 signaling on CRC pathogenesis. While IL-1R deletion in epithelial cells blocks CRC progression independent of inflammation, the same defects in the T cells and the myeloid cells (mainly neutrophil) restrict and exacerbate tumor growth and progression, respectively, following the microbial invasion in tumors (104). The targeted ablation of source bacteria can block the compensatory loop. Similarly, in the mice inflammation model, CD4-driven IL-10 production through macrophages augments IL-17 production (105). Fn is a dominant player in this paradigm. It drives a shift that augments formate production. Aryl hydrocarbon receptor (AhR) signaling promotes invasion and cancer stem cell properties in *in vitro* co-culture of Fn with CRC cells under this condition. In mice, Fn injection increases the Th17 cell expansion and tumor growth (106). Since IL-17 low MMRp tumors favor ICB outcome, similar cross-talk, and context are expected to persist in their suppressive TME. The γδT-17 cells represent another subset that drives an MDSC bias in CRC (67, 107, **Figure 2**).

### 4.3 Diversity and metabolites influencing MMRp milieu

Systems-level diversity of the gut microbiome describes their influences in shaping CRC tumor niche. The 16S rRNA gene sequencing of dMMR (n=29) and pMMR(n=201) in tumors (T) and matched adjacent normal (N) tissues deciphered critical differences in their diversity both at alpha and beta levels. Overall, species diversity of gut microbiome (alpha diversity) was higher in the MMRd-T niche than in MMRp-T and MMRd-N. This comparative profiling showed significant differences (beta diversity) between MMRd and MMRp (**Figure 2**). Secondary Kyoto Encyclopedia of Genes and Genomes (KEGG) pathway analysis confirmed microbiota-related glycan metabolism, vitamins and nucleotide biosynthesis, active cell death, and defects in DNA repair machinery in MMRd tumors. Indeed, these properties favored PFS and OS upon immunomodulator exposure in MMRd and outperformed MMRp, where microbiota relies predominantly on lipid metabolism (108).

In a permissive MMRd ecosystem, microbiota supports vitamin A metabolite and retinoic acid accumulation. It galvanizes nucleic acid and protein breakdown machinery in a heterogenous gut immune-interaction network. The accumulation of lactate and other short-chain organic acids like propanoic acid, owing to microbiota depletion in MMRp, makes the tumors immunosuppressive (109) (**Figure 2**). Lactic acid-producing bacteria *Lactobacillus iners* rewire host tumor metabolic pathway in cervical cancer and confer chemo- and radiotherapy resistance. A similar L-Lactate producing bacterial population reduces recurrence-free survival (RFS) in colorectal adenocarcinoma (110). Targeting the metabolic hardwire that regulates local oxygen levels or reduces hypoxia will provide insights into their therapeutic prospects (111, 112). For example, antagonists targeting immune-specific CD73 or genetic deletion of A2AR, reversing lactate and hypoxia-induced immune suppression are in clinical development (113, 114). Therefore, combining such agents with approved immune or non-immune therapies may boost the anti-tumor response in MMRp. A spatial metabolomics landscape of the tumors also adds an interactive milieu in this context (115). Extending its crosstalk with the microbial metabolomic network will further define the metabolic vulnerabilities in MMRp and other similar tumors.

### 4.4 Ecosystem deep mining in MMRp precision microbiome

While the proximal and distal gut microbiota define the fate of tumors, intra-tumoral bacteria in such scenarios pose a serious health challenge. Integrated metagenomics and metabolomic profiling further expand the scope of ecosystem-level deep mining and shed light on undetected metabolites (116, 117). Pan cancer profiling of intra-tumoral bacterial hubs helped elucidate their distinct indication-specific composition. It also confirmed their intracellular presence, which covers tumor and immune cells. For example, Firmicutes and Bacteroidetes phyla were the two most abundant species in a cohort of 22 CRC samples (118). From these perspectives, the distinct orientation of dysbiosis and its polymorphic microbiome spectrum are suspected to drive toxin-induced mutagenesis in the gut ecosystem (119). CRC patient-derived fecal gavage has been associated with inducing GI tract carcinogenesis in germ-free mice (120). Contrary to this, CRC with MMRp persuasively displayed a hostile metabolic microenvironment that facilitates disease progression and therapy resistance. The conceptual progress in tumor-microbiome interactions also sheds new light on the presence of intertumoral bacteria and their geospatial micro-niche in the tumor ecosystem. New single-cell RNA sequencing technology has mitigated the low biomass challenge and improved the robustness of capturing the tumor microbiome diversity. The preferentially high bacterial population density in vasculature deprived (i.e. CD34 negative) and Ki67 negative pockets with suppressive immune contexture in CRC forms the basis for the non-random heterogeneity of microbiota (121). This profiling added valuable knowledge about resources in such tumor ecosystems and can gauge potential benefits from complementary therapy.

Intra-tumor microbiome maps of CRC (and GI) have been developed in a pan-cancer study. It revealed an MSI-MSS distinction of their communities. It also showed poor survival after ICB in the *F. nucleatum* high group in the case of NSCLC (109, 122). However, there is a paucity of knowledge on the premises of MMRp (102, 118). As a dysregulated immune-microbiome interplay in MMRp CRC corrupts TME, reinstating a patient-friendly microbiota context and relevant therapeutic strategies potentiate a better therapy response in MMRp (109, 122, summarized in **Figure 2** and **Table 2**). Further studies on the intracellular bacterial population in MMRp+ CRC and its spatial distribution in an immune-excluded context coupled with metabolic programming will offer valuable insights and perspectives. The comprehensive landscape of tumor-associated bacteria highlights the scope of fine-tuning therapeutic intervention at the tumor-microbiome interface.

**Table 2:** Ongoing clinical trials that include MMRp positive CRC patients.

Clinical Trial ID /Trial Name	Phase, size (N), Status	Cancer Type	Agents	Targets	Design and end points measure
NCT04457284	Phase II (N=18) Active, not recruiting	MMRp Colorectal Adenocarcinoma	Temozolomide Cisplatin Nivolumab	PD-1	Single Arm, Open label Primary endpoint: Response (RECIST)
NCT05609656	Phase II (N=12) Recruiting	mCRC	Device: Irreversible electroporation Device: Calcium electroporation Pembrolizumab	PD-1	Single Arm, Open-label Primary end point: rate of AE as per CTCAE v4.0 Secondary endpoints: Tumor response by CT and USG, PFS, OS Other endpoints: immune infiltration by CD3, CD4, CD8, PD-1 and PDL-1
NCT03519412 (ARETHUSA)	Phase II (N=102), Active, not recruiting	MMRd mCRC, MMRp CRC (MGMT negative by IHC)	Temozolomide (induction), Pembrolizumab	PD-1	Non-randomized, Parallel, Open-label Primary endpoints: ORR (RECIST v1.1, iRECIST) Secondary endpoints: PFS, OS, safety and tolerability
NCT05160727	Phase II (N=44) Recruiting	MMRp/MSS inoperable recurrent/mCRC	Tislelizumab Irinotecan Radiotherapy	PD-1	Single Arm, Open-label Primary endpoint: ORR
NCT05870800	Phase II (N=30) Not Recruiting	Stage I, II and III MMRp Colon Cancer	Tecentriq + Capecitabine + Oxaliplatin, Oxaliplatin + Leucovorin + 5-Fluorouracil, Oxaliplatin + Capecitabine	PDL-1	Single Arm, Open-label Primary endpoints: ORR, CR or PR, Secondary endpoints: RFS, ctDNA change (pre-, mid-, post-NAT), AE, QOL (PRO)
NCT05980689	Phase II (N=33) Recruiting	MMRp/MSS locally advanced Rectal Cancer	AK104 Capecitabine Neoadjuvant Radiotherapy	PD-1-CTLA4 (bispecific ab)	Single Arm, Open label Primary endpoints: CR, pCR, cCR Secondary end points: AE, DFS, OS
NCT03711058	Phase I/II (N=48) Active, not recruiting	Unresectable or Metastatic MSS Solid Tumors along with MSS Colon Cancer, Colon Cancer	Copanlisib (phase I) Nivolumab (phase II)	PI3K, PD-1	Non-randomized, Sequential, Open-label Primary endpoints: DLT, ORR, PR, CR by RECIST 1.1 Secondary endpoints: DCR, DOR, PFS, OS
NCT05205330	Phase I/II (N=28) Active, not recruiting	Refractory mCRC, Solid Tumor, Metastatic MSS CRC, MMRp CRC	CR6086 (Phase I) AGEN2034 (Phase II)	EP4 receptor, PD-1	Non-randomized, Single Arm, Open-label Primary end points: Safety, tolerability, DCR (CR, PR, SD) Secondary endpoints: ORR, CR, PR by RECIST/iRECIST), DOR, PFS, OS, TEAEs
NCT05933980 (REGOTORICOX)	Phase II (N=44) Recruiting	CRC: Liver Metastases, MSS	Regorafenib+ Toripalimab +Celecoxib	VEGFR 1–3, PD-1	Single Arm, Open label Primary endpoint: ORR Secondary endpoints: OS, PFS, DCR, DoR
NCT03851614 (DAPPER)	Phase II (N=90) Active, not recruiting	MMRp-CRC, Pancreatic Adenocarcinoma, Leiomyosarcoma	Durvalumab Olaparib Cediranib	PDL-1, PARP, VEGFR	Randomized, Parallel, Open label Primary endpoints: Base line and On Tx genomic and immune markers Secondary endpoints: ORR, CBR, PFS, OS, Response (RECIST), AEs
NCT04724239	Phase II (N=48) Active, not recruiting	Advanced MSS CRC, MSS CRC	Sintilimab Chidamide IBI305	PD-1, HDAC, VEGF-A	Randomized, Parallel, Open-label Primary end point: PFS (18 weeks) Secondary end points: ORR, PFS, OS, DCR, DoR
NCT05609370	Phase I/II (N=226) Recruiting	Unresectable or Metastatic MSS/MMRp CRC	LBL-007 Tislelizumab Bevacizumab biosimilar Capecitabine 5-Fluorouracil	LAG3, PD-1, VEGF-A	Randomized, Parallel, Open-label Primary endpoint: ORR (RECIST v1.1) Secondary endpoints: OS, PFS, ORR, DOR, Cmax of LBL-007 (for Phase I only)
NCT02060188	Phase II (N=385), Active, not recruiting	MSI CRC, MSS CRC, MMRp CRC, MMRd CRC	Ipilimumab Nivolumab Cobimetinib Daratumumab BMS-986016	CTLA-4, PD-1, MEK1, CD38	Non-randomized, Parallel, Open-label Primary endpoint: ORR (RECIST v1.1) Secondary endpoints: ORR by RECIST v1.1 by IRRC
NCT06356597	Phase II (N=25) Recruiting	MSS/MSI-L advanced CRC with high abundance of *Fusobacterium nucleatum*	Tislelizumab with Fruquintinib, Metronidazole	LAG3, VEGFR-1,-2, and -3	Single Arm, Open-label Primary endpoint: ORR
NCT05733611	Phase II (N=4) Active, not recruiting	Refractory mCRC, MMRp, MSS	RP2 RP3 Atezolizumab Bevacizumab	4–1BB and CD40 PDL-1, VEGF-A	Non-randomized, Parallel, Open-label Primary endpoint: ORR Secondary end points: TEAEs, SAEs, OS, PFS, DoR, DoCB, CCR
NCT03712943	Phase I (N=52) Active, not recruiting	CRC, mCRC, Colon Cancer	Regorafenib, Nivolumab	VEGFR1–3, TIE2, PDGFR-β, FGFR, KIT, RET, RAF, PD-1	Non randomized, Sequential, Open label Primary end point: MTD Secondary endpoints: RR (RECIST), OS, SAE

mCRC, Metastatic Colorectal Cancer; MSI, Microsatellite Instability; MSI-L, Microsatellite Instability-Low; MSS, Microsatellite Stability; MMRd, Mismatch Repair-deficiency; MMRp, Mismatch Repair-Proficiency; CR, Complete Response; pCR, pathological Complete Response; cCR, clinical Complete Response; PR, Partial Response; AE, Adverse effects; ORR, Objective Response Rate or Overall Response Rate; DFS, Disease-Free Survival; OS, Overall Survival; PFS, Progression-Free Survival; DCR, Disease Control Rate; DoR, Duration of Response; MTD, Maximum Tolerable Dose; RR, Response Rate; RECIST, Response Evaluation Criteria in Solid Tumors; SAE, Severe Adverse Event; CBR, Clinical Benefit Rate; iRECIST, immune related RECIST; RFS, Relapse-Free Survival; ctDNA, circulating tumor DNA; NAT, Neoadjuvant Therapy; QOL, Quality of Life; PRO, Patient Reported Outcome; Cmax, Plasma Maximum Concentration; TEAE, Treatment Emergent Adverse Event; IRRC, Independent Radiology Review Committee; DoCB, Duration of Clinical Benefit; CTCAE, Common Terminology Criteria for Adverse Events; CT, Computed Tomography; USG, Ultrasonography. For further details of all the trials listed here, please refer to ClinicalTrials.gov (https://www.clinicaltrials.gov).

### 4.5 Therapeutic strategies in reversing onco-microbiome niche in MMRp

Several strategies leveraged diverse aspects of microbiomes and their perturbation in preclinical and clinical settings and identified their potentials and limitations.

#### 4.5.1 Probiotic gut bacteria in metabolic immunomodulation in MMRp

The probiotic bacterium *Clostridium butyricum* inhibits Wnt signaling, reduces the risk of colon cancer development and boosts anti-microbial macrophage function while sparing inflammation-induced tissue damage (123, 124). Faecal microbiota analysis of CRC patients revealed that *Roseburia intestinalis*, a probiotic species, and the metabolite (butyrate) generated by it, protect mice (CT-26 and MC 38) and human hosts from gut inflammation and damage. They also unleash CD8-induced anti-PD-1 efficacy in MSI-low/MMRp (CT-26) orthotopic mice. The substantial depletion of this species was observed in patient-derived stools compared to healthy individuals. Its transfer to mice from healthy humans inhibited tumor growth (125). A conversation between Group 3 innate lymphoid cells (ILC3s) and T cells through the engagement of MHCII complex following supplementation of microbiota orchestrate type 1 innate immune response and responsiveness to PD1 inhibition in mice. Lack of MHC-II in ILC3s or microbiota harvested from ILC3s dysregulated subjects failed to elicit immune therapy response following their transfer to mice (126).

#### 4.5.2 Microbiome as an adjuvant in immune checkpoint therapy

Similar systemic clearance was reported when Fn was targeted by silver nanoparticle-bound M13 phage (Ag-M13). Reduction of Fn-induced MDSC and reinvigoration of APC functions were reclaimed under this therapy condition in mice models. Ag-M13 acted in synergy with ICB or chemo-agents (127). In general, antibiotics also have precedence in interfering with ICB (128). The dose, sequence, spectrum, limited and emergency-only use of antibiotics can overcome this challenge. Target-specific antibiotics and bacteriophages make their way to better deal with this situation (66, 129). Through modulating specific chemokine production, gut microbiota helps infiltrate the anti-tumor T cells to the tumor sites and improves the survival opportunities (130). Bifidogenic live bacterial products complementing the microbiome, tested initially in renal cell carcinoma (RCC), would add an interesting vertical in this direction (131). More importantly, several vaccines targeting Fn and Bf in colon cancer are under preclinical development. Depending on the risk association, these vaccine candidates will be used for therapeutic or prophylactic purposes (132).

#### 4.5.3 Microbiome guided therapy

Analysis of drug-metabolome association in allogeneic hematopoietic cell transplant (alloHCT) recipients among cancer patients may benefit these populations. Longitudinally tracked fecal microbial species revealed a substantial loss of alpha diversity or dysbiosis. It also gathered information regarding a reversal under different medications. An *in silico* computational prediction model mirrors the *in vitro* measurement of antibacterial activity and patient clinical outcomes (133).

Antibiotics can reduce bacterial loads that pose a threat to prognosis and response to therapy. For example, *F. nucleatum* in CRC accumulates succinic acids. This high succinic acid in tumor hinders response to PD1 inhibition by obstructing CD8 cells. Both FMT from the responder and antibiotic metronidazole overcome this restrain (66). Likewise, by enhancing the safe and effective local delivery, liposomal antibiotic administration in mice targeting *F. nucleatum* elicited cytotoxic T cell response through increasing the immunogenic neoantigen burden of bacterial origin. This modulation further helps in T cell priming and recognition of antigen-naive and reactive tumors (134) (**Figure 2**). In clinical CRC, before surgical resection, eliminating anaerobic bacteria load upon antibiotics treatment improved disease-free survival (DFS) by 25.5%.

Antibiotics can lead to the vertical loss of healthy intestinal flora, but probiotic and fecal microbial transplantation (FMT) can compensate for the antibiotic-induced loss of gut microbiota. Current limitations within this realm involve compatibility, stability, unknown composition, kinetics, and dynamics, which could be ethical concerns. For an amenable resolution of these concerns, instead of adopting a blanket use, individualized assessment of gut health and other supporting methods like physical activities are important factors that can improve the outcomes (135–137). Reimposing anti-dysbiotic barriers requires coordinated approaches. Supplementing the gut ecosystem with niche-modifying commensal species prevents colonization by invaders. It prevents the accumulation of metabolically challenging pathogenic microbes and releases bacterial antigens to boost the pro-immunogenic immune network (137) and **Figure 2**.

## 5 Response prediction biomarkers for improving therapy outcomes in MMRp

Both immune and targeted therapy rely on the individualized selection of patients to maximize benefit from a given treatment modality. Integrating biomarkers that predict response is of pivotal importance in this context. This also provide information pertaining to resistance and help designing rational therapeutics.

### 5.1 Immune response in MMRp and mechanistic underpinnings

Collectively, 85% of CRCs are of MMRp type (138). Overall, 10% CRC and 5% metastatic CRC of MMRp status show a response to ICB (33). Combining anti-PD1 with novel anti-CTLA4 in heavily pretreated (median prior line: 4) CRC with MSS status showed promising safety and efficacy (ORR 24%). In case of no history of liver metastases/ablation of liver metastases without recurrence, a better outcome was achieved (n=24, ORR 42%, and DCR 96%). This response included a patient with SD (RECIST 1.1) who showed ongoing metabolic complete response (mCR) by PET after CEA normalization. For all responder cases, metastatic sites spanned soft tissue, peritoneum, retroperitoneum, pleural effusions, bone, lungs, and lymph nodes. Responder mutation profile confirmed *RAS* mutations (4 *KRAS*, 1 *NRAS*), no *BRAF* mutations, a high TMB (TMB=10) in one case, one case of CPS >50%, and no single *POLE* mutations cases (139). Both MSS CRC with and without liver metastasis showed benefits from ICB, where liver metastasis conferred more frequent resistance (140). This implies the urgent requirement to improve the overall response to this therapy in MMRp tumors, reducing its gap with MMRd. Indeed, challenging the current response rate for all modalities with new and more effective treatment regimens is a continuous process and needs innovative, rational approaches integrated with multimodal diagnostics and predictive tools.

#### 5.1.1 IL-17 and LAG-3 are therapy barriers

From oncoimmune perspectives, however, response predictive gene signatures revealed that a preexisting immunoreactive profile does not explicitly depend on suppressive tumor immune microenvironment represented by spatial CD8 and IFN-γ and colocalized PDL-1/IDO1 checkpoint genes. Irrespective of IL-17 low or high niche states, the IL-17 low MMRp landscape mimicked a primary CRC responsive to ICB. In the same study, a panel of immunomodulatory genes (precisely, *LAG-3, CD8A, CD4, CD274*) showed similar expression patterns between MMRp and MMRd responder cohorts. However, it indicated reciprocal downregulation in the MMRp non-responder cohort (67). Rationally targeting the IL-23/Stat3/IL-17 signaling axis in IL-17 high MRRp+ CRC may offer a mechanistic basis for overcoming adaptive resistance to ICB. Analysis of TCGA data and cell line profiling of MMRp from CRC revealed that high expression of immunoglobulin superfamily 6 (*IGSF6*) is correlative with infiltration of CD4+ T cells, CD8+ T cells, CD68+ macrophages and conferred sensitivity to immunotherapy and chemotherapy (141). Lineage tracking elucidated an interesting new role of CD4 cells in both providing help to cytotoxic CD8 cells and directly acting as cytotoxic killer cells. This observation unlocks a new gate for understanding its implication of targeting coinhibitory receptor LAG-3 that mechanistically crosstalk with MHC class II (142–145). Interestingly, a first-in-human multicohort safety and efficacy study of anti-LAG-3 antibody MK4280 (favezelimab) with pembrolizumab in CRC that progressed on two prior lines following combination (2C+5), ORR was 6.3% (4PR, 1CR by RECIST). In contrast, the median duration of response (DOR) and OS were 10.6 months and 8.3 months, respectively. Both these endpoints were better compared to monotherapy. In particular, patients with PDL-1 status >1 combined positive score (CPS) showed a prominent response (146).

Beating the current ORR across therapeutic modalities in MMRp is a formidable challenge. It warrants smart and novel vulnerability mapping strategies. There is an increasing interest in understanding ostensibly dysfunctional immune contexture. Fine-tuning the T cell pre-exhaustion dynamics is critical for preventing their final differentiation into terminally exhausted T (TET) cells (147, 148). TET cells present an irreversible phenotype and frustrate immune intervention strategies like PDL-1 blockade. For poorly immunogenic MMRp tumors, targeting other potentially actionable MMRp and non-MMRp vulnerabilities would exert similar mileage. New therapeutic developments leveraging new immunomodulators are in the preclinical pipelines to potentiate this paradigm shift (**Table 1**).

### 5.2 DNA damage repair pathways in MMRp targeted intervention

**Figure 3:**
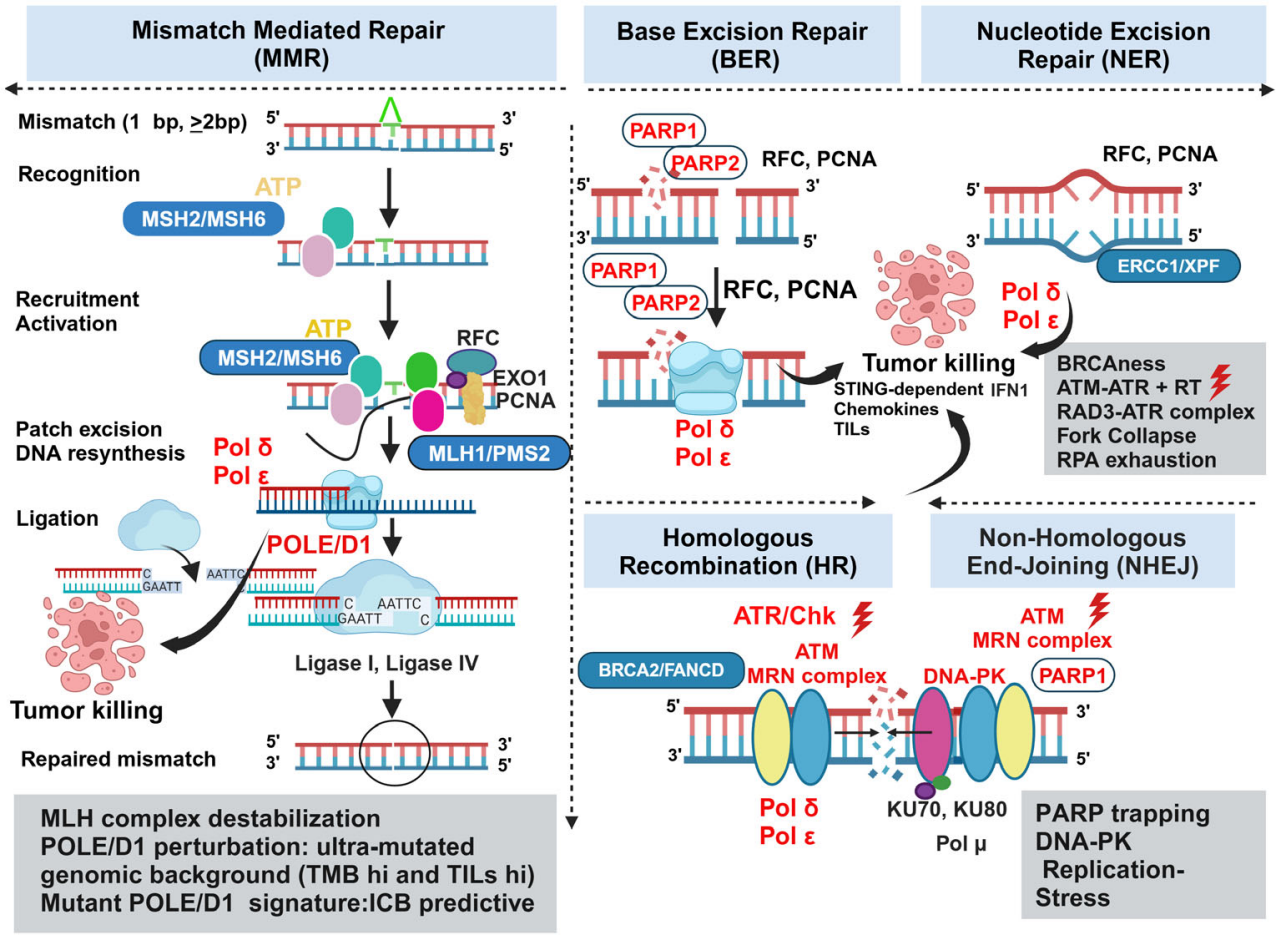
Vulnerabilities and alternative actionabilities in MMRp tumors decipher the key biomarkers and molecular targets in DDR machinery. MMRp tumors efficiently bypass key base pair mismatches using a repair mechanism that recruits repair proteins in the recognition-activation-resynthesis-ligation cascade. Although MLH complex destabilization and PLOEed perturbation are key actionable areas, the limited options in this class of MMR system highlight the need to search for parallel alternative targets involved in base excision repair (BER), nucleotide excision repair (NER), homologous recombination (HR) and nonhomologous end joining (NHEJ). Biomarkers for each repair category are presented in boxes. Therapeutic targets and their perturbations are indicated in red. POLE/D1, PARP, ATM, ATR, and Chk are key targets for which drugs are either under clinical development or approved for one or more indications. ATR and concurrent radiation can act in synergy to induce STING-dependent IFN1 production, and deliver conditional lethal hits leading to the killing of the tumors. Key steps of molecular mechanisms are depicted in the figure and mentioned in corresponding boxes. MSH, MutS homologs; MLH, MutL protein homolog; PMS2, Postmeiotic segregation Increased 2; EXO1, exonuclease 1; RFC, Replication factor C; PCNA, proliferating cell nuclear antigen; POLε, DNA polymerase epsilon POLδ, polymerase delta; XP-F, xeroderma pigmentosum; ERCC1, excision repair cross-complementation group1; FMCD2, Fanconi anemia group D2; MRN, Mre11-Rad50-Nbs1.

DNA damage response (DDR) as an overtly orchestrated system has multiple actively operating networks like class 1 defects in double-strand break (DSB) and replication repair (*BRCA 1* and *BRCA 2* mutations), class 2 defects in signaling (ATM, ATR, CHK1, CHK2) and class 3 defects (MMR) leading to high TMB (81). Understanding the therapeutic opportunities of targeting each of these defects in colorectal cancers has gained momentum in recent years. Several modalities targeting them are under active development (149). Dissecting the diverse facets of counter-regulation and coregulation of their interactive molecular circuits in governing the protection of the tumor cells against cytotoxic insults offers novel opportunities for turning the MMRp tumors vulnerable to emerging therapies (**Figure 3**).

#### 5.2.1 POLE/D1 at the center stage of MMRp targeted intervention

Pathogenic missense mutations in proofreading enzyme polymerase epsilon (POLE) - at DNA binding and catalytic sites, largely operating in gastric cancer and CRC, have been implicated in perturbing MMR efficiency and generated ultra-mutated genomic landscape illuminated with both high TMB and TILs predictive for ICB response. From these perspectives, POLE/D1-like molecules provide a unique example of alternative and complementary target biology and raise the hope of benefiting MMRp patients to ICB (150). Mice CT26 tumors harboring Pole P286R mutant clones showed better response to ICB mono and combination therapies by a 3-fold increase in CD3 infiltrations. However, they extended modest survival in the patient tumor-derived xenograft (PDX) model. These may be due to the absence of human-specific stable immune contexture in PDX. A mutant POLE/POLED1 signature outperforms traditional approaches in stratifying patients likely to benefit from ICB. These data further suggest that a pathogenic mutation affecting the fidelity of DNA repair enzyme can boost anti-tumor immunity of ICB (151).

#### 5.2.2 Vulnerability beyond MMRp: spotlight on the alternative ATM-ATR axis

Nonredundant and alternative DNA repair pathways spatially and temporally converse and converge to avoid human replication protein A (RPA) exhaustion followed by “replication catastrophe” and cell death. Ataxia telangiectasia and Rad3 related (ATR) protects the cells from this vulnerability (152, 153). In contrast, their defects lead to synthetic lethality. BRCA 1 and BRCA 2 are two targets for which there is interest in developing biomarker-guided DNA repair agents. These agents perturb classical non-homologous end joining (NHEJ) and other complementary repair systems like homologous recombination (HR) and alternate end joining (alt EJ). Molecules that leverage the target biology of PARP, Ataxia-telangiectasia mutated (ATM), ATR, CHK1 and WEE 1 are either in trials or under development (154) and **Figure 3**. Study showed that concurrent radiation and inhibitor of ATM, a DNA damage repair protein, elicits tumor growth inhibition mainly by augmenting STING-dependent IFN1 production and chemokines critical for immune infiltration. In CT 26 and MC38 mice models, ceralasertib, a potent ATR inhibitor, showed no direct effects on tumor killing, which is typical for this class of agents. Instead, it induces immunomodulating effects on proliferating CD8+T cells when intermittent dosing was applied in contrast to continuous dosing. It changes monocyte-MDSC (M-MDSC) and TAM dynamics and increases DC in mice TME. Type 1 IFN (IFN1) is augmented in cancer patients upon ceralasertib therapy (155). Therapy-induced upregulation of PDL-1 and MHC1 on the tumor surface further offers a temporal window of sequential PDL-1 inhibition in combination with anti-ATM agent (156). Although this study was done in the mice model of HNSCC, it perceivably reciprocates the same mechanism of action in the MMRp-like context where poor immunogenicity is a confounding factor. Preclinical studies using multiple *in vivo* mice models deciphered the involvement of ATR-mediated DNA repairing machinery in radiation-resistant CRC. This defect impaired DC-mediated tumor antigen cross-presentation via upregulation of CD47 (‘eat me not signal’) and PDL-1. It drives further crosstalk through the cognate engagement of PD-1 and SIRPα signaling cascade. A rationale combination of RT with anti-SIRPα and anti-PDL-1 targeting this axis resulted in a complete response in primary and abscopal tumors in a STING-dependent manner. These data imply the mechanistic link between ATR inhibition in inducing anti-tumor response when the DNA repair pathway confers RT resistance (157). ATR- ATR-Checkpoint Kinase 1 (Chk) also surged as a viable target aiming to overturn MMRp-driven therapy constraints. DNA alkylating agent MNNG induced MeG/T mismatch lesion by inhibiting Chk1 signaling. *N*-methyl-*N′*-nitro-*N*-nitrosoguanidine (MNNG) orchestrated the fork collapse and DSB in embryonic stem cells in the absence of ATR-Chk1 activation. It also perturbed their ability to handle replication stress and led to rapid induction of apoptosis. However, a transient S phase checkpoint in Hela cells under MNNG pressure and the active state of ATR-Chk1 induced G2 arrest (158).

#### 5.2.3 Targeting DNA-PK in MMRp

Another critical barrier that obstructs successful therapy outcomes is linked to chemotherapy (CT) and RT-induced DNA damage. Subsequent evasion of this response by a compensatory repair mechanism is mediated by the DNA-dependent protein kinase (DNA-PK). Further dissection of this network revealed that both NHEJ and HR could happen sequentially. In that case, DNA-PK and MRN/CtlP coordinate in this event (159). The open-label, phase I trial of peposertib (formerly M3814), an inhibitor of DNA-PK, showed tolerance in a cohort of 31 solid tumor patients. However, only modest outcomes (stable diseases) were observed in 12 patients for >12 weeks (160). Nevertheless, the targetability of DNA-PK has been established in multiple *in vitro* preclinical studies, including studies that demonstrated the druggability of its catalytic domain subunits using small molecule inhibitors (161). Learning from molecular biology harnessed DNA-PK mediated excessive end resection to the non-propagating quiescent G0 phase. However, it was not evident in the G1 or G2 phase of the cell cycle owing to the detachment of FBXL12, a ubiquitylation-promoting factor that targets KU70/KU80 subunits of DNA-PK only in G0 (162) and **Figure 3**.

#### 5.2.4 Unlocking the potential of epigenetic targets in MMRp

In addition to the germline and somatic coding mutations in key MMR enzymes, transcriptional silencing of MLH1 through promoter hypermethylation (MLH1 methylation) was observed in 10–20% of all CRC cases. This MLH1 methylation is one of the main causes of sporadic CRC (162). Promoter hypermethylation in hMLH1 gene is associated with microsatellite instability and BRAF mutations, accompanied in some cases by somatic loss of the wild-type allele (163, 164). However, the status and impact of MLH1 methylation are less explored in MMRp from a translational perspective (27). Epigenetic readers, writers and erasers/degraders represent an active cluster for therapeutic development. Their roles in MMRp tumors of diverse indications still need to be fully elucidated. N6-methyladenosine (m6A) METTL3/METTL14, the writer constituents of methyltransferase complex (MTC), impeded TILs recruitment in MMRp CRC. Targeted silencing of this axis augmented STAT1-mediated IFN-γ production and elicited anti-tumor effects (165). Analysis of TCGA data, tissue microarray, RNA-Seq and preclinical mice experiments using MC38 (MDSC rich), CT26 (immune inflamed, MMRp), and CD34/immune reconstituted humanized immune CRC xenograft mice models deciphered that m6A reader YTHDF1 had an inverse correlation with IFN-γ gene signature. Indeed, perturbation of YTHDF1 by gene silencing averted resistance to anti-PD1 therapy by inhibiting MDSC infiltration and boosting cytotoxic CD8 functions in MMRp-positive CRC (166).

#### 5.2.5 DDR targets in MMRp: challenges and path forward

As DDR based therapeutics are gaining rapid momentum in the targeted oncology arena, there are outstanding challenges related to their target biology validation, structure-based drug design and selectivity. Multiple DDR targets have high sequence homology. For instance, DNA-PK, ATM and ATR share similar sequences; therefore, there is more likelihood of off-target effects. Cryo-electron microscopy (Cryo-EM) enables structural resolutions of ATM and ATR. However, it needs other coactivating proteins like RPA for ATR and MRN for ATM. This knowledge gap currently hinders structure-based DDR drug design (167). Mechanistically, when more than one repair modalities operate, they tend to diminish the efficacy of the selected agent. Besides redundancy and limited biomarkers for target specificity selection, toxicity, target loss and target resistance are leading drivers of efficacy loss. ATM and ATR axis can be used as salvage therapy in PARP inhibitor refractory tumors or expanded for HRP tumors (168). Uncertain actionability with limited knowledge of the microenvironment context also poses a challenge. Some targets, like *BRCA* mutations, have a low prevalence (5%) in CRC, mostly confined to MSI-H. Even in *BRCA* mutant cancers, the tumors can escape inflammation-driven immune attacks using lesser-known mechanisms that are both tumor-intrinsic and tumor-immune microenvironment-regulated in nature (169). Although PARP inhibitors are at the forefront of DDR driven therapies, PARP-trapping by proximity ligation assay in *BRCA*1 mutant breast cancer (CaBr) showed both efficacy and off-target bone marrow toxicity following PARP inhibitor monotherapy and poses a challenge for combination (170). The recent withdrawal of late-line PARP inhibitors for multiple indications warrants further learning of the root causes (171). For all these targets, pharmacological dosing and additional mechanisms of actions involving immune-mediated and direct killing are not elaborated in most of the investigations. Similarly, there is scope to gain more insights into whether ATR at continuous dose is inferior to intermittent (holiday) doses.

## 6 Emerging diagnostic tools for defining precision medicines in MMRp

The clinical CRC world is equipped with robust selection biomarkers like *KRAS* mutations and MSI/MMR. Current clinical guidelines recommend testing the MMR status of all CRC samples irrespective of the clinical stages of the disease. PDL-1 genetic variation (del, polysomy, amplification) is more frequent in MMRd compared to MMRp in new CRC at the time of diagnosis and is linked to poor prognosis (172). High congruence (99%) of MMRp proteins was observed between IHC and MSI molecular testing based on a large sample size of >3K and inter-site cross-validation (173). Discordance was observed when samples were collected using different methods; sample volumes varied, and different training methods were used to handle the tissues (174. Pancreatic and endometrial cancers with Lynch Syndrome (LS) were the two areas of high discordance between MSI and MSH (175). An AI-guided classifier achieved a performance score that appreciated the clinical benchmark (95% sensitivity for MMRp/MSS and MMRd) without taking help from any manual annotation steps (176). MMRp and MMRd binary paradigm has been shifted in recent years (27). For an ongoing process like MMR, a robust, specific and sensitive assay incorporating multiple inputs and its validation will reduce the false detection of MMRd due to the unrelated presence of high TMB.

### 6.1 Liquid biopsy in treatment management of MMRp-specific indications

Liquid biopsy (ctDNA) has emerged as a valuable tool to longitudinally monitor treatment outcomes or tumor progression non-invasively. In patients with advanced GI cancers, ctDNA accelerated enrolment (doubled compared to regular biopsy) by shortening screening duration three times without any negative impact on treatment outcomes (177, 178). Moreover, clonal landscaping from 2000 patient-derived liquid biopsy samples identified several actionable driver alterations (177). Further, a study of 445 CRC patients with stage 2 disease (2:1 randomization) evaluated liquid biopsy-guided management. A ctDNA-positive result at 4 and 7-weeks post-surgery prompted a chemotherapy decision. In contrast, patients who were ctDNA-negative were spared from CT. This study elucidated that ctDNA-guided 2 years of recurrence-free survival was non-inferior to non-ctDNA-guided standard clinicopathological criteria (93.5% and 92.4%, respectively; 95% CI, -4.1 to 6.2 (noninferiority margin, -8.5 percentage points). These data indicate the promise of this approach in managing adjuvant chemo treatment without enhancing the risk of recurrence-free survival (179).

#### 6.1.1 Liquid biopsy as an integrated tool in precision multi-omics

**Figure 4:**
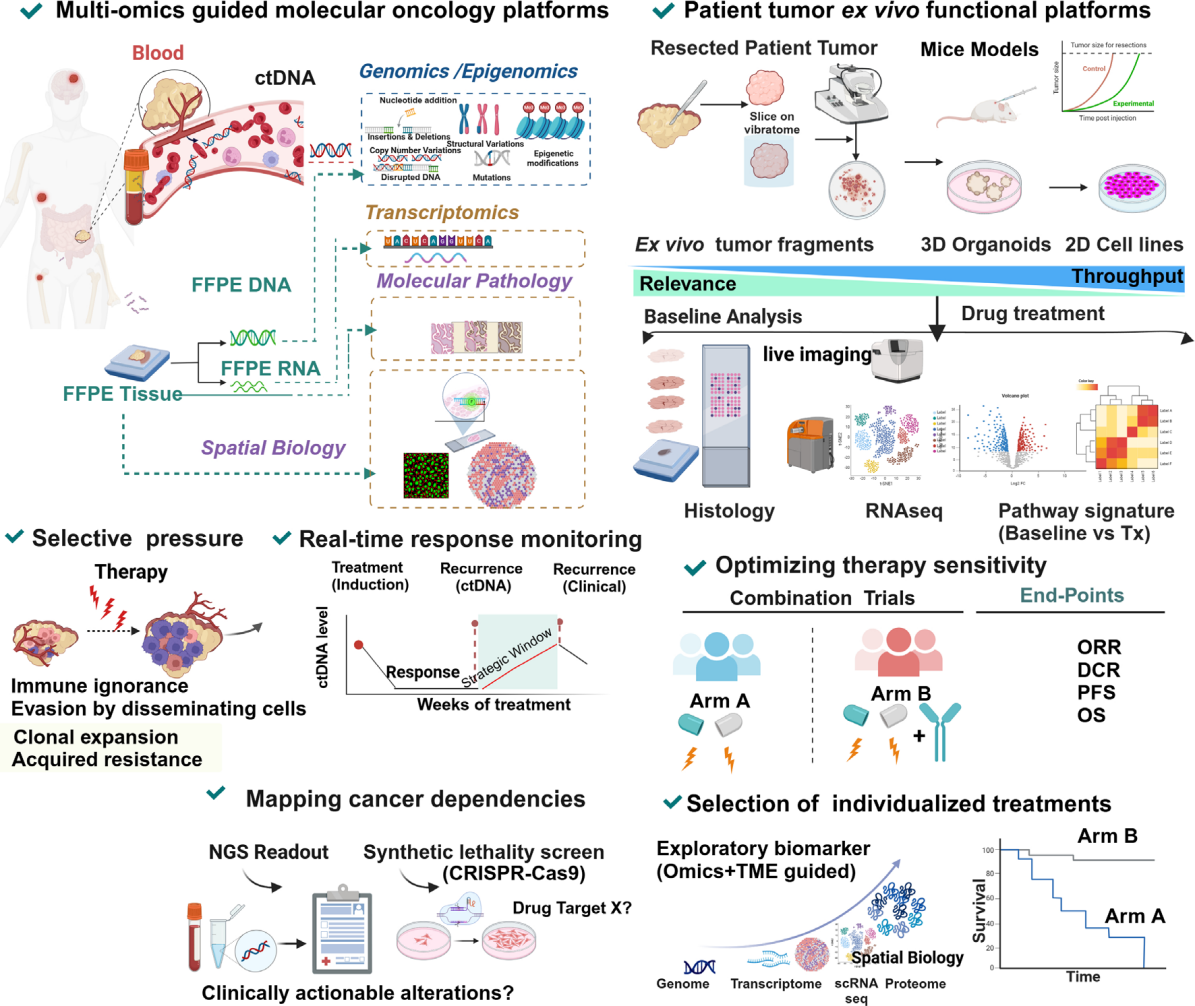
Precision oncology molecular multi-omics and functional platforms in predicting recurrence, response and guiding rational combinations in MMRp. Information obtained from systematic and multilayered molecular profiling of patient tumors converging genomics, epigenomics and proteomics from a longitudinal analysis of liquid biopsy and clinical biopsied samples (fresh unfixed or fixed tissues) provide critical spatiotemporal dynamic contexts of biomarkers, signatures and tumor-immune interface. Finally, it helps predict the recurrence risk, including therapy-driven or therapy-independent recurrence and clonal expansion. Change in ctDNA levels in serum is a reliable predictor that informs about a prospective clinical recurrence and, therefore, opens a strategic window in guiding the treatment plan ahead of recurrence. Multiple synthetic lethality screens like CRISPER knockout and conditional lethality decipher the pathway dependency and oncogenic addictions for delineating the druggable targets (180). Functional prediction platforms led by microenvironment-guided drug sensitivity screens actively leverage information from contextually relevant phenotypic readouts in a mechanistic setting. This clinical avatar works in coordination with molecular oncology modalities where clinically meaningful evidence of actionability is available and can provide an alternative solution when such biomarker information is absent or not translatable. An integrative cross-functional approach uses multiple live systems covering 2D cell lines, 3D organoids and non-dissociated tumor slices depending on the requirements and availability. Mice models can still evaluate the systems-level modulation of drugs and their synergy. These models show the advantage of obtaining data from real-world diverse assays using live cultures focusing on drug reactivity and functional modulation patterns in time and space. The provision is there to integrate the outputs into a predictive score. The clinical relevance, speed and scalability are not uniform across the platforms. Microenvironment-guided selection of optimal therapy combination in trials led by such assay outputs takes informed decisions by integrating multi-omics and spatial biology context at single-cell levels. The platform-guided selection has the power to improve response rate and differentiate superior combinations and synergy.

As illustrated in **Figure 4**, liquid biopsy can be used as an alternative or complementary tool along with other multi-omics platforms like functional genomics, epigenomics and spatial biology insights in a systems biology context to a) predict response and a potential relapse in the clonally biased immune evaded TME, b) expand the strategic window to combat clinical recurrence and, c) provide a synthetic lethality screen to underpin clinically actionable drug targets for these advanced, primary treatment failure conditions (181–184).

These approaches harmonize in guiding critical treatment decisions for naïve, relapsed or refractory conditions and therefore open new trial/therapy opportunities where regular tissue biopsy is not feasible or there is an urgent need for molecular guided (e.g. in CRC, *KRAS*/*NRAS*/*BRAFV600E*/MSI) treatment decisions (177, 185). An earlier systematic review in 2018 highlighted a relatively low clinical actionability that has remained a bottleneck for decades in selecting NGS-guided rationale therapies. It benefits only a minor (10%) percentage of patients representing indications like NSCLC, melanoma and RCC from the frontline ICB (186). However, recent findings from the MSK-IMPACT assay that used OncoKB 2017 and 2022 versions showed overall evidence-driven improvement of clinical actionability for existing SOCs and IO agents. The platforms increased the enrolment in clinical trials for new agents. Likewise, the actionability scaled from 8.9% to 31.6% (187). This study also exposed the gaps where non-actionable alterations (i.e., cases where no response predictive or treatment selection biomarkers are available) are confined mainly to TP53 (43%), *KRAS* (19%) and *CDKN2A* (12.2%). More importantly, only MSI-H and TMB high showed a rise in the actionability trajectory.

For non-actionable, more precisely, cases where no drug-matching biomarkers are available, liquid biopsy-based *ex vivo* functional filters can directly inform drug sensitivity (188). Indeed, ctDNA-based approaches do not readily provide critical spatial biology insights during diagnosis and treatments and are not ready to replace conventional biopsy.

Further validation of liquid biopsy through clinical trials may save time and resources in late-phase development by its informed integration for DDR *ATM*, *BRCA 1,2* related mutation profiling (189). CTCs outbound for a clustered migration have a much higher potential to metastasize than solitary CTCs. Intercellular compartments (nanolumenal) concentrating growth-promoting ligands facilitate high metastatic potential (190). Phenotypes or molecular signatures that differentiate the CTC-derived oligoclonal precursors open new avenues to tailor next-generation precision medicine solutions in relevant landscapes.

## 7 From platforms to patients: functional predictive tools for MMRp driven therapy

Success and failure of oncology and immunooncology drug trials hinge on three main verticals- a: profound insights into target biology and anti-target ‘avoidome’, b: preclinical platforms, and c: time of the decision to go for the trials (191). The upfront limitation of preclinical platforms is that the models that filter a drug for late-stage nominations are highly porous due to a lack of critical contexts mechanistically mirroring a patient’s tumor immune microenvironment. The traditional *in vitro* and *in vivo* mice tumor models remain the backbone of discovery research for decades. Recent *in vitro* cell line panels helped identify potential combinations based on targeted drug screens for CRC and other indications (192). Similarly, mice data demonstrated the feasibility of parallel response modeling of multiple agents in tandem to accelerate this screening phase (193). However, in I-O, their independent contributions are not consistent. This constraint also limits the biomarker-guided patient selections. These perspectives prompted the development and validation of other alternative platforms that can reduce dependency on conventional systems if not completely replaced. Integrating molecular signature and spatial TiME contexts adds powerful predictive insights (194) and **Figure 4**. They also minimize the trial failure rates. PDX, syngeneic mice and their *ex vivo* 3D culture counterparts, when integrated and complemented with multiplex readouts, could help advance our understanding of metastatic diseases and bridge the critical knowledge gaps (195). Reverse translation of mice data and forward translation of the short-term *ex vivo* data synergistically may augment their predictive power of immune checkpoint response (196).

### 7.1 Patient-derived 3D tumor models as predictors of response dynamics

In recent years, patient-derived 3D organoids, organ-on-chips, and non-dissociated tumor fragments have emerged as functionally relevant platforms to screen drugs in TME settings that are close to the real world (**Figure 4**). They guide prioritizing novel combinations based on specific vulnerabilities (197). Studies showed that these models can be adopted rapidly. They can faithfully predict ICB drug reactivity and clinical outcomes based on parameters and scores generated using the assays and their discriminatory contributions (198, 199) and **Figure 4**. Two independent utility studies highlighted that *ex vivo* response prediction can signal a positive correlation in the case of liquid cancers. Kornauth et al. reported an image-based single-cell functional precision medicine (scFPM) n-of-one approach. In 54% of cases it showed >1.3-fold enhanced PFS after median follow-up for 23.9 months. Exceptionally, 40% of the responders showed a three-times longer duration of response than expected. A second study using a multi-omics *ex vivo* platform in a functional precision medicine tumor board (FPMTB) reported 97% actionability. This study also reported 59% ORR and linkage of IL-15 overexpression with resistance to FLT3 inhibitor and instructed to alloHSCT for 5 patients (200, 201). A recent *ex vivo* study with 101 bone marrow samples from 79 eligible patients further informed on the variability of the drug sensitivity. It defined patient stratification based on actionable multiplex pathology inputs and image-based deep learning (202). CRC-driven models are well represented in this new era of *ex vivo* technology (65, 155, 203–206) and **Figure 4**.

In CRC, peritoneal metastasis is associated with the lowest survival rate. Therapies that can improve the OS are limited for this condition. Narasimhan et al. described a medium-throughput *ex vivo* ‘peritoniods’ model for addressing this challenge (207). The model showed a 68% (19/28) success rate of stability. Most patients whose ascites were used were prediagnosed with the worst prognosis CMS 4/MSS. Drug sensitivity testing using this model led to a decision impact on two patients. One of the patients who had multiple rounds of treatment failure showed partial response (PR) to the gemcitabine–capecitabine combination arm three months post-therapy. Notably, regorafenib failed to show sensitivity against any sample (207). The observed lack of response to regorafenib may be due to compromised angiogenic and stromal contexts in *ex vivo* ascites (207–209). EGFR inhibitor osimertinib and HDAC inhibitor vorinostat also displayed higher sensitivity. However, regulatory barriers to off-label therapy prohibited testing them on the patients. Another patient received ‘off-label’ Vandetanib on compassionate grounds, but it was too late to attain the expected benefit. These findings demonstrated the value of ascites-based *ex vivo* organoids in informed treatment selection in clinically challenging scenarios.

### 7.2 Translation of *ex vivo* data in molecular and phenotype-guided prediction

Although not all *ex vivo* platforms are high throughput, they provide suitable substrates to test multiple drug arms in parallel. In evaluating five drug combinations in parallel, an *ex vivo* organotypic slice culture identified potential benefits only from the combination of Src inhibitor and MEK inhibitor. In 29 surgically resected samples from MMRp CRC in this study, the baseline level of phosphorylated Src was used as a biomarker coupled with wild-type *KRAS* G12 (210). Mechanistically, an independent study delineated the consequence of inhibiting the RAF-MEK-ERK axis. The death of *KRAS* mutant CRC organoids was observed upon exposure to low doses of RAF and ERK inhibitors in combination (211). A similar screen may help prioritize rational synergy in MMRp CRC. The microorganospheres (MOS) derived from CRC patient biopsy enabled rapid testing with a turnaround time of 8–14 days. Moreover, testing the immunotherapy agent in this MOS predicted sensitivity with 75% accuracy and resistance (NPV) with 75% accuracy (212). Encouraging results were obtained from the co-culture of tumor-immune organoids of CRC from MMRd and MMRp backgrounds. This study differentiated the CD8-driven killing of CRC organoids upon ICB. Further elucidation of this response revealed the selective engagement of MHC-I and cytotoxic effector molecules (203). Indeed, this study independently predicted better efficacy of ICB in MMRd associated CRC and lent credence to this platform for systems-level testing of similar IO modalities and preserving MoA (**Figure 4**). The realization of *ex vivo* response modeling is gaining momentum, particularly after the inspiring outcomes from dostarlimab trial in CRC from MMRd class (213). It galvanizes the efforts of looking for similar benefits in other cancers.

### 7.3 Patient-mirrors of functional phenotypes and genomic fidelity

Unlike cell lines of primary origins and, to some extent, patient-derived organoids and PDX, short-term explant slices largely retain genomic fidelity without introducing any new driver or pathogenic variants (214–218). In this context, RNAseq data demonstrated intra and inter-tumor variability in the retention of clonal and immune landscapes in GBM. Despite underlying heterogeneity, patient baseline and corresponding non-dissociated tumor explant fragments matched better than their primary cell line counterparts (219). Metabolically, *In vivo* and PDEx models demonstrated the preferential utilization of glucose over L-glutamine. This background gives these models an edge over cell line-based *in vitro* culture (220). Indeed, metabolic heterogeneity in cancer is a dynamic paradigm and depends on factors that regulate local oxygen and nutrient gradients, tumor cell density, stromal composition, exosome dynamics, and ECM stiffness. In temporal settings, therapy-induced changes and metastasis also adapt altered metabolic programs (17, 19).

PDEx maintains angiogenesis gene signature and blood vessel phenotypes in *ex vivo* culture (205, 221). However, their functional impact still needs to be elucidated. Hasselluhn et al. demonstrated the active maintenance of critical cell types such as tumor epithelial, fibroblast, myeloid, T cells and blood vessel density in short-term *ex vivo* culture. In this system, the paracrine cascade of hedge hogde (HH) activation promoted angiosuppression by inhibiting WNT and VEGFR2 signaling. Inhibition of Smoothened (SMO) in this background induced angiogenic switching. Subsequently, the explants regained vascular level hypersprouting following inhibition of the HH pathway target (222). Experimental evidence also supports the ability of the organoids and tumor fragments to gauge radiosensitivity. Organotypic brain slice culture (OBSC) using a multiparametric algorithm led to developing a sensitivity score that normalizes off-target effects (223). Besides retaining the tumor, immune and genomic fidelity, efforts to recreate or retain microbial interface in *ex vivo* culture have also recently gained momentum (224).

### 7.4 *Ex vivo* 3D platforms offering diverse cross-functional and multimodal readouts

The deliverables of *ex vivo* response prediction rely on a battery of complementary, multimodal and cross-functional assays and integrated scores (**Figure 4**). Real-time monitoring of drug reactivity in PDEx system needs quantitative evaluation using baseline and proper vehicle control to capture optimal assay peak and corresponding delta. An integrative model of preferably label-free spatial live imaging, in contrast to terminal snapshots, complementary cross-functional bioassays, multiplex spatial omics at baseline and on treatments, adds more edges to the robustness and multimodality perspectives in the current systems (225–227). As tumor agonistic approaches, these functional tools rapidly gain momentum in late oncology drug developments and leverage forward and reverse translation for rationale combinations. In previous studies, the number of samples with matched clinical outcomes was either indirect or limited. Further trials aim to understand the clinical utility of these functional screening platforms (228). The decision to co-culturing tumor with immune compartments like PBMC and TILs must be carefully informed based on the target indications and candidate drugs. For greater acceptability, current limitations of *ex vivo* models, like variability, must be overcome (229, 230) and **Table 3**. Best practices for tissue procurements for IO trials are evolving. Similar guidance can be implemented to improve *ex viv*o prediction (231). New regulatory guidelines have classified the *ex vivo* systems as laboratory-developed tests (LTD) and medical devices (MD). Therefore, these tests are under the high-risk category and subject to more stringent validation and quality control requirements (228, 232).

## 8 Discussion and future direction

CRC is a challenging late-stage neoplasm for which there is a pressing need to develop more effective therapies. This review delves into MMRp pathophysiology, current progress in its complex molecular landscape, immune contexts, metabolic state and biomarkers. These are the major pain points for cracking the therapy barriers and beating the overall low response rate of the existing drugs. Transformative approaches are vital to overturn the global mortality rates tied to MMRp. About 1–10% of MMRp is MSI-H. They maintain high TILs compared to the MMRp-MSS group and resemble MMRd (27). Cross-learning from MMRd enriches our knowledge. Enhancing the scope of adaptive therapy for MMRp tumors appreciates strategies undertaken for MMRd cancers (233). The MSI-MMRp subgroup with high TILs is uniquely positioned in this spectrum.

Deciphering the unique TiME in MMRp tumors and addressing its low TMB is crucial for rational renormalization. However, the key obstacles include a restrained anti-tumor immune paradigm, a deficient or depressed TILs-TLS state, and an overwhelming TGF-β/Th17 signaling bias that explicitly fosters immune suppression (234). MMRp TiME is highly decorated with tumor-promoting chemokines and cytokines networks presented by CXCL 1–8, CXCR4, IL-10 and their divergent downstream cascades. They choreograph the tumor-homing and retention of the immunosuppressors like monocytes, TAN, TAM and Treg cells (62) (**Figure 1** and **Table 1**). Capturing a comprehensive chemokine footprint can help rejuvenate immune modulation and suppress tumor-promoting functions. Multiple strategies are emerging to reconstruct the chemokine network. Small molecules and antibodies to target pro-tumor ligands, oncolytic virus-expressing chemokine ligands and neoadjuvant vaccines are few that have raised optimism (59, 60), (**Figures 1**, **2**; **Table 1**).

We discussed the impact of a skewed gut microbiome on treatment resistance and immune evasion. Changes in microbiome diversity leads to an environment high in lactate and other tumor-protecting metabolites (66, 109, 110). This microbiome-immune cross-talk disrupts immune cell function through TGF-β and Th17 signaling, and hinders key immune activation cascades. We illustrated the strategies to combat this hostile TiME (66) (**Figures 1**, **2**; **Tables 1**, **2**). The critical insights gained from these approaches will shape future research and clinical practices.

Indeed, the gut microbiome poses promises and, at the same time, personalization challenges in treating CRC. The abundance of *F. nucleatum* in CRC impairs ICB effects on CD8 through local succinate build-up (66). Eliminating tumor-invading *F. nucleatum* and other toxicogenic species and restoring healthy microbiota showed early promise across MMR classes (66, 68, 127, 129, 132, 134). However, safety and efficacy data from more extensive trials is needed before routinely adopting this modalities. Several studies demonstrated the untapped potential of molecularly alternative vulnerabilities. Targeting specific enzymes like POLE/D1 and other complementary MMR-independent repair mechanisms like ATM/ATR/DNA-PK and PARP may offer new treatment avenues in this direction (**Figure 3**).

Resources are emerging to prioritize molecular oncology precision through leveraging omics and multi-omics platforms, including liquid biopsy for response monitoring in advanced CRC (189). This can enhance actionable omics-guided predictive biomarkers. In heterogeneous and dynamic settings like CRC, genomics data alone may not be enough to determine therapy efficacy in the MMRp context. Complementary approaches like liquid biopsy and TME-guided functional platforms can be stand-alone options and conform to positive changes in trials and management. *Ex vivo* tumor models like 3D organoids, slices, and ascites showed the potential to understand therapy resistance and optimal treatment decisions for CRC (197, 198, 203, 235). Insights from spatial biology integrated with AI/ML open a new frontier in personalized combination selection (**Figure 4**; **Table 3**). Contrasting responses to ICB in MMRp and MMRd have been demonstrated using CRC-derived organoids (203). Studies are investigating the predictive strength of *ex vivo* models (65, 228, 236). However, the current status indicates that further clinical validation and refinement are essential before the widespread adoption of these functional screens. Space limit prevents detailed discussion of all the subtopics here.

**Table 3:** Key strengths and limitations of precision oncology molecular and functional platforms.

Liquid biopsy and NGS: Promises in MMRp critical indications	Functional oncology platforms in MMRp critical indications
Enables periodic monitoring of the diseases Faster (3X) recruitment in trials with same safety and efficacy Provides important biomarker information ( e.g. *KRAS*/*NRAS*/*BRAF-V600E*/*MSI*/*MSS*) Fast-tracks the optimal therapy decision in real-time Helps in sparing unwanted chemo-regimens Noninferior for RFS prediction Informs TMB status Delineates the mutant/ resistant clones Identifies the driver alterations (e.g. mutations in *ATM*, *BRCA1,2*) Can be integrated with other complementary approaches Useful when visceral biopsy is a challenge Can help in *ex vivo* drug sensitivity testing	**Mice models:** Good for general understanding at the level of discovery research Heterotopic and orthotopic sites are not mirrored Syngeneic mice model: Poor IO driven forward translation PDX, humanized PDX: Good for targeted agents and MoAs Human immune context is not near baseline. Good for comparing cold with hot Long time and high cost to develop, clonal landscape different than patient
	**2D In vitro (cell lines):** HTP screens, suitable for chemo and targeted therapy Not suitable for IO, attrition is high Contextual porosity makes it a weak model
**Limitations in MMRp** Not yet ready to replace biopsy Critically spatial TiME context is missing Genomics and phenotypes matching is not possible Not all cancers and patients have an actionable alteration(s)	**Ex vivo Slices, Organoids, Organ-on-Chips:** Offer reasonable throughput, provisions for MoA customization and personalization PDEx, and to some extent, organoids maintain near baseline (native) TME TiME and proteogenomic fidelity are better represented Compatible for spatial biology context and microbial interface analysis High degree of actionability (>70%), integration with biomarker is possible Provide discriminative scores and outcomes for IO and non-IO, diverse therapies Angio-suppression, off-target based normalization possible. Encouraging clinical prediction value in both solid and liquid indications
	**Limitations of ex vivo functional platforms:** Heterogeneity and variability in response need additional strategic layers Short-term culture. Organoids take months to develop, success rate varies Lack of active blood vessels limits testing drugs directly working on this MoA Biopsies and visceral samples from metastatic sites are difficult to collect Prior exposure to therapy affects quality of test materials Inter-assay, inter-lab harmonization and thresholding is not well-defined

Ongoing trials pave the way for exploring rational combinations in MMRp, often with promising results. For example, a triple combination of PD1 inhibitor, HDAC inhibitor, and anti-VEGF agent significantly improved PFS and ORR. RNA sequencing confirmed high CD8 infiltration in the triplet arm cohort (237). Neoadjuvant DC vaccine boosted the immune profile (60). Other ongoing trials that leverage combinatorial opportunities in MMRp-positive CRC are summarized in **Table 2**. A collaborative framework that continues to engage clinical experts and translational scientists on this premise will reduce the time required to develop new therapeutic modalities. Finally, the outlook evolving from ongoing MMRp research sets to transform the journey toward precision oncology.

## Author contributions

BM: Conceptualization, Writing – original draft, Writing – review & editing, Visualization. LM: Writing – review & editing. NN: Writing – review & editing. SD: Writing – review & editing.
